# Mincle and STING-Stimulating Adjuvants Elicit Robust Cellular Immunity and Drive Long-Lasting Memory Responses in a Foot-and-Mouth Disease Vaccine

**DOI:** 10.3389/fimmu.2019.02509

**Published:** 2019-10-29

**Authors:** Min Ja Lee, Hyundong Jo, Sung Ho Shin, Su-Mi Kim, Byounghan Kim, Hang Sub Shim, Jong-Hyeon Park

**Affiliations:** ^1^Animal and Plant Quarantine Agency, Gimcheon-si, South Korea; ^2^Gyeonggi Veterinary Service Laboratory, Yangju-si, South Korea

**Keywords:** PRR ligands, cytokines, adjuvants, foot-and-mouth disease, vaccine

## Abstract

Conventional foot-and-mouth disease (FMD) vaccines exhibit several limitations, such as the slow induction of antibodies, short-term persistence of antibody titers, as well as low vaccine efficacy and safety, in pigs. Despite the importance of cellular immune response in host defense at the early stages of foot-and-mouth disease virus (FMDV) infection, most FMD vaccines focus on humoral immune response. Antibody response alone is insufficient to provide full protection against FMDV infection; cellular immunity is also required. Therefore, it is necessary to design a strategy for developing a novel FMD vaccine that induces a more potent, cellular immune response and a long-lasting humoral immune response that is also safe. Previously, we demonstrated the potential of various pattern recognition receptor (PRR) ligands and cytokines as adjuvants for the FMD vaccine. Based on these results, we investigated PRR ligands and cytokines adjuvant-mediated memory response in mice. Additionally, we also investigated cellular immune response in peripheral blood mononuclear cells (PBMCs) isolated from cattle and pigs. We further evaluated target-specific adjuvants, including Mincle, STING, TLR-7/8, and Dectin-1/2 ligand, for their role in generating ligand-mediated and long-lasting memory responses in cattle and pigs. The combination of Mincle and STING-stimulating ligands, such as trehalose-6, 6′dibehenate (TDB), and bis-(3′-5′)-cyclic dimeric guanosine monophosphate (c-di-GMP), induced high levels of antigen-specific and virus-neutralizing antibody titers at the early stages of vaccination and maintained a long-lasting immune memory response in pigs. These findings are expected to provide important clues for the development of a robust FMD vaccine that stimulates both cellular and humoral immune responses, which would elicit a long-lasting, effective immune response, and address the limitations seen in the current FMD vaccine.

## Introduction

Foot-and-mouth disease (FMD) is a highly contagious viral disease that mainly affects cloven-hoofed livestock. This disease causes serious economic losses to the livestock industry, due to a rapid spread and high livestock mortality, resulting in low livestock productivity (1). Over 70 species of wildlife, including livestock ruminants such as cows, pigs, buffalos, camels, sheep, and goats, are susceptible to this disease. FMD is associated with high fever and causes blisters on the mouth, tongue, snout, nose, nipple, hoof, and other hairless areas of the skin (2).

Immunization with inactivated vaccines, which are used as a means of controlling the disease in countries afflicted by FMD, constitutes an important part of the contingency plans drawn up to meet emergency situations in FMD-free countries (3). Similar to other vaccines that were generated against viral diseases, several trials designed to generate a live attenuated vaccine for FMDV have failed due to unstable phenotypes, variable pathogenic profiles, risk of virus transmission, and failure to induce adequate protection (4). Inactivated vaccines are used against FMD globally. In order to produce an effective vaccine, an antigen purification process, which removes cellular contaminants as well as non-structural viral proteins (NSPs), is required to facilitate diagnostic testing that differentiates infected animals from vaccinated animals (DIVA). Since vaccine antigens consisting of dead viruses do not replicate or induce antibodies against NSPs, anti-NSP antibodies have often been used as markers of infection. The efficacy of inactivated vaccines was improved by including oil adjuvants (double or single oil emulsions). However, these vaccines exhibited certain limitations, such as the slow induction of antibodies to levels allowing for defense, low antibody titers, short-term persistence of antibodies, and low immunogenicity in pigs.

FMD vaccines focus on inducing humoral immune responses rather than cellular immune responses. But their protective effect is not perfect. While the period taken for induction of the humoral immune response, via the major neutralizing antibody, IgG, by FMD vaccines is 4–7 days (5), T cell-mediated cellular immune response is generated by innate immunity, where injection of Ag or infection with a pathogen activates innate immune cells within a few hours to 2–3 days (6). These cells then trigger inflammatory responses by secreting proinflammatory cytokines, chemokines, and costimulatory molecules. This innate, cell-mediated, immune response is amplified within 3 days and peaks after 3–7 days. It is an effective defense system that can recognize and clear the virus rapidly in the early stages of FMDV infection or reinfection. Moreover, current FMD vaccines have a short duration of antibody persistence following inoculation, requiring periodic vaccinations at intervals of 4–6 months. When intramuscularly administered to pigs in particular, these vaccines often cause lesions, such as fibrosis and granuloma in the inoculated muscles, indicating issues such as local side effects and low safety. Although studies related to FMD-related vaccines have focused on investigating the efficacy of vaccines in cattle, rather than in pigs, the immunogenicity induced by vaccination is lower in pigs than in cattle (7). Therefore, to overcome the limitations of the current commercial vaccines, the ideal vaccine design should have the following characteristics: simultaneous induction of both cellular and humoral immune responses, maintenance of high antibody titers through the induction of memory response, achievement of safety to reduce local side effects, and a new strategy for the development of adjuvants optimized for different livestock species.

Various adjuvant-related studies have investigated methods for improving protection against FMD, including an evaluation of the efficacy of FMD vaccines in pigs and goats using pattern recognition receptors (PRR) ligands such as Resiquimod (R848), poly(I:C) (8), muramyl dipeptide (MDP), monophosphoryl lipid (MPL), and β-glucan (9). Use of immune-boosting agents such as rapeseed oil and ginseng root saponin (10) as well as commercially available adjuvants such as ISA 201, ISA 206, Emulsigen-D, and Carbigen have also been evaluated. However, thus far, adjuvant-induced perfect immunity has not been found. In an effort to increase immunogenicity, focus was placed on the induction of cellular and humoral immune responses, as well as human vaccines rather than FMD vaccines, and the following were studied and utilized as adjuvants (11, 12): (1) vaccine delivery systems such as oil emulsions, surfactants, liposomes, virosomes, and immune-stimulating complexes; (2) immune-boosting agents such as saponin, aluminum hydroxide (Al(OH)_3_), and potassium phosphate; (3) receptor-specific immune stimulators such as Toll-like receptors (TLRs), RIG-I-like receptors (RLRs), nucleotide-binding oligomerization domain (NOD)-like receptors (NLRs), and ligands for C-type lectin receptors (CLRs); and (4) a variety of cytokines such as IL-1, IL-2, IL-6, IL-18, TNFα, IFNγ, and GM-CSF. Some of these are currently in use or undergoing clinical trials for use as vaccine adjuvants for the prevention and treatment of various human diseases such as cancer, tuberculosis, hepatitis B, malaria, influenza, human immunodeficiency virus, and the herpes simplex virus (13, 14), but none have been utilized as a component of FMD vaccines. Moreover, since different adjuvants have different modes of action, it is important to understand the immunological mechanism underlying the role of these adjuvants in order to facilitate the development of FMD vaccines using a new strategy that may induce strong cellular and humoral immune responses simultaneously.

Our group conducted intensive studies to investigate different serotypes of FMDV Ag-mediated cellular immune response *in vivo* and *in vitro* (murine, bovine, and porcine immune cells) as well as the effectiveness of various PRR ligands and cytokines as adjuvants in mice. We also examined their ability to induce cellular and humoral immune responses in mice and analyzed related mechanisms to elucidate the differences in immune responses among livestock species, such as cattle and pigs. Therefore, in order to develop specific adjuvants optimized for each livestock species and produce novel FMD vaccines that included these adjuvants, this study pursued the following objectives: evaluate memory response induction by adjuvants, including PRR ligands and cytokines; screen adjuvants that stimulate immune responses in peripheral blood mononuclear cells (PBMCs) isolated from the whole blood of cattle and pig; evaluate the composition of the experimental vaccines, including adjuvants selected for their ability to induce a humoral immune response *in vivo* (cattle and pigs); propose a new strategy for the development of FMD vaccines.

## Materials and Methods

### Antigen (Ag) Purification and Inactivation

Ags were prepared by cultivating the FMD virus (FMDV) O/TWN/97-R (GenBank AY593823 for P1) in BHK-21 cells according to the method described by Lee et al., with modifications (15). To initiate viral infection, the culture medium was replaced with serum-free Dulbecco's modified Eagle's medium (DMEM; Cellgro, Manassas, VA, USA), and the cells were inoculated with the virus and incubated for 1 h at 37°C in a 5% CO_2_ atmosphere. All extracellular viruses were then removed. At 24 h post-infection, the viruses were inactivated with two treatments of 0.003 N binary ethylenimine for 24 h in a shaking incubator (16) and concentrated using polyethylene glycol (PEG) 6000 (Sigma-Aldrich, St. Louis, MO, USA). The virus concentrate was layered onto 15–45% sucrose density gradients and centrifuged (17). After ultracentrifugation, the bottoms of the centrifuge tubes were punctured and 1 ml fractions were collected. The presence of FMDV particles in a sample of each fraction was confirmed by optical density using a lateral flow device (BioSign FMDV Ag; Princeton BioMeditech, Princeton, NJ, USA). Prior to its use in the experiment, the pre-PEG treatment supernatant was passed through ZZ-R and BHK-21 cells at least twice to ensure that no cytopathic effect (CPE) occurred, thereby confirming the absence of any live viruses in the supernatant.

### PRR Ligands and Cytokines

PRR ligands were purchased from InvivoGen (InvivoGen, San Diego, CA, USA), and cytokines were purchased from Mitenyi Biotec (Miltenyi Biotec, Bergisch Gladbach, Germany) and R& D Systems (R&D Systems, Minneapolis, MN, USA). ISA 206, an oil emulsion, was purchased from Seppic Inc. (Paris, France), and aluminum hydroxide gel (Alhydrogel® and Quil-A were purchased from InvivoGen.

### Mice

Age- and sex-matched wild-type C57BL/6 mice (7-week-old females) were purchased from KOSA BIO Inc. (Gyeonggi, Korea). All mice were housed in microisolator cages in a specific pathogen-free animal facility at biosafety level 3 (ABSL3) at the Animal and Plant Quarantine Agency. The studies were performed according to institutional guidelines and with approval from the Ethics Committee of the Animal and Plant Quarantine Agency.

### Memory Immune Response Mediated by PRR Ligands and Cytokines in Mice

**Figure 1:**
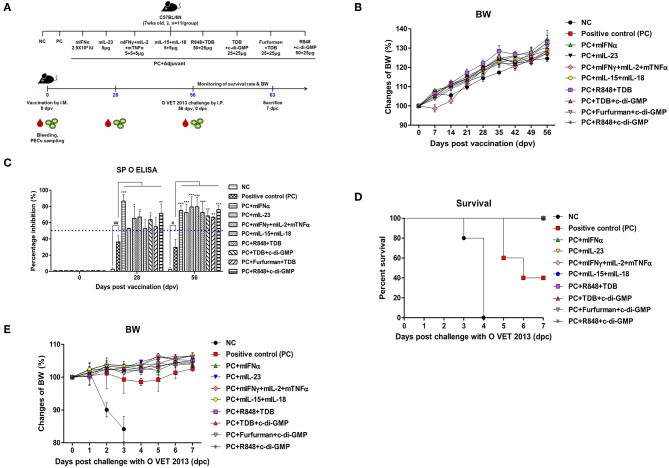
Adjuvanticity of PRR ligands and pro-inflammatory cytokines; significant enhancement of the memory response in mice against FMDV infection C57BL/6 mice were administered either a combination of PRR ligands or cytokines with the vaccine based on the vaccine composition of the positive control group. The PRR ligands and cytokines used in the experiment were as follows: TDB (Mincle agonist), c-di-GMP (STING agonist), Furfurman (Dectin-2 agonist), R848 (TLR-7/8 agonist), mIFNα, mIL-23, mIFNγ, mIL-2, mTNFα, mIL-15, and mIL-18. A negative control group of mice was injected with the same volume of PBS as the vaccine, and a positive control group received 11.7 ng (1/160 of the dose for cattle or pig use) of O/TWN/97-R Ag, ISA 206 (50%, w/w), 10% Al(OH)_3_, and 15 μg Quil-A without PRR ligands and cytokines. The test vaccines were injected intramuscularly into mice that were later challenged with FMDV (100 LD_50_ O VET 2013) at 56 dpv. Blood sampling was performed on 28 dpv and 56 dpv for the serological assays. The survival rates and body weights were monitored for 7 dpc. **(A–E)** represent **(A)** the strategy for this study; **(B)** antibody titer by SP O ELISA; **(C)** changes in body weight post vaccination; **(D)** survival rate against FMDV (O VET 2013); **(E)** changes in body weight post challenge with O VET 2013. The data are the mean ± SEM of triplicate measurements; statistical analyses were performed using two-way ANOVA with Bonferroni correction; ^#^, *p < 0.05, ^*##*^, **p < 0.01, and ***p < 0.001.

To evaluate the potential of PRR ligands and commercially available recombinant cytokines as vaccine adjuvants, and to investigate their protective effect against FMDV infection and their ability to induce a memory response, experiments were performed using the strategy shown (Figure 1A) (*n* = 11 per group). O/TWN/97-R Ag was used as inactivated FMDV Ag. The vaccine composition for the positive control (PC) group was as follows: O/TWN/97-R Ag (15 μg/dose/ml, 1/160 dose), ISA 206 (50%, w/w), 10% Al(OH)_3_, and 15 μg/mouse of Quil-A for a total volume of 100 μl. All experimental group mice received vaccines with the same composition as the PC group, with the addition of either PRR ligands or recombinant cytokines as an adjuvant. Mice in the negative control group received an equal volume of phosphate-buffered saline (PBS, pH 7.0) administered via the same route. Briefly, the mice were vaccinated intramuscularly in the thigh muscle. Later, 56 days post vaccination (dpv), the mice were challenged with FMDV (100 LD_50_ of O VET 2013, ME-SA topotype) by intraperitoneal (I.P.) injection. Their survival rates and body weights were monitored up to 7 dpc. In addition, serum and peritoneal exudate cells (PEC) sampled from mice at 0, 28, and 56 dpv were analyzed via structural protein enzyme-linked immunosorbent assay (SP ELISA), virus neutralization (VN) titers, and PEC subpopulations to determine the ability to induce cellular and humoral immune responses. In order to identify whether FMDV O Ag-specific T cell responses and memory T cell responses were amplified by Ag re-stimulation, we isolated pre (0 dpi) and post Ag injection (28 dpi) mice PEC (PC group). T cells were purified from isolated PEC (Pan T Cell Isolation Kit II, Miltenyi Biotec) and sorted via flow cytometry (purity > 98%). T cells were cultured at 37°C and 5% CO_2_ in complete RPMI media (Gibco, Carlsbad, CA, USA) supplemented with 10% FBS (HyClone, Logan, Utah, USA), 10 mM HEPES (Gibco), 10 U/ml penicillin/streptomycin (Sigma-Aldrich), and 50 mM 2-mercaptoethanol (Sigma-Aldrich). Cells were subsequently treated *in vitro* with or without Ag (1 μg/ml) for 6 h. The percentage of IFNγ^pos^CD4^+^ T cells and IFNγ^pos^CD8^+^ T cells was compared via flow cytometry as described in 2.5. ELISA for IFNγ (R&D Systems, Minneapolis, MN, USA) was also performed on T cell culture supernatants according to the manufacturer's instructions.

### Flow Cytometric Analysis

In order to analyze PEC subpopulations, single-cell PEC suspensions (0.5–1 × 10^6^ cells in PBS supplemented with FBS) were incubated with purified anti-CD16/32 antibodies (Abs) (FcγRII/III block, Clone. 2.4G2; eBioscience, San Diego, CA, USA) to block non-specific staining. PEC was immunostained with fluorochrome-conjugated Abs to CD3 (Miltenyi Biotec, Clone. REA641), CD4 (Miltenyi Biotec, Clone. REA604), CD8a (Miltenyi Biotec, Clone. 53-6.7), CD44 (Miltenyi Biotec, Clone. REA664), CD62L (Miltenyi Biotec, Clone REA828), CD27 (Miltenyi Biotec, Clone. REA499), anti- γδ TCR (Miltenyi Biotec, Clone REA633), CD335 (NKp46) (Miltenyi Biotec, Clone. REA815), CD11c (Miltenyi Biotec, Clone REA754), anti-MHC Class II (Miltenyi Biotec, Clone. REA813), CD11b (Miltenyi Biotec, Clone REA592), and anti-F4/80 (Miltenyi Biotec, Clone. REA126). For intracellular staining of cytokines, cells were stimulated by PMA and ionomycin in the presence of Golgi-stop (BD Bioscience, Franklin Lakes, NJ, USA) in complete RPMI medium for 4 h. After stimulation, cells were washed, and surface molecules were stained. Cells were then fixed with Intracellular (IC) Fixation Buffer (eBioscience), washed with Perm Buffer, and stained with anti-IFNγ (Miltenyi Biotec, Clone. REA638). Data were acquired via flow cytometry (MACSQuant® Analyzer 10, Miltenyi Biotec) and analyzed by FlowJo software vX 0.7 (TreeStar, Ashland, OR, USA). Cell counts were performed in duplicate following the addition of Trypan blue dye using a Vi-CELL Series Cell Viability Analyzer (Beckman Coulter, Brea, CA, USA).

### PBMC Isolation

Bovine and porcine whole blood was donated by the Gyeonggi Veterinary Service Laboratory. FMD antibody-seronegative animals were used as donors (*n* = 4/group for bovine PBMC; *n* = 6/group for porcine PBMC). Whole blood (15 ml/each donor) was independently collected in a BD Vacutainer heparin tube (BD, Becton, Dickinson and Company, Franklin Lakes, NJ, USA), and PBMCs were isolated using Ficoll-Paque™ PLUS (GE Healthcare Bio-Sciences Corp., Piscataway, NJ, USA) gradient centrifugation. Residual red blood cells were lysed by treating them with ACK (ammonium-chloride-potassium) lysing buffer (Gibco, Carlsbad, CA, USA). The PBMCs were suspended in Dulbecco's PBS without Ca^2+^ and Mg^2+^ (Gibco, Carlsbad, CA, USA), supplemented with 2% fetal bovine serum (FBS) (Gibco, Carlsbad, CA, USA), and counted using a volumetric flow cytometer (Miltenyi Biotec). All cells were freshly isolated directly before use, and no cryopreserved cells were used in any experiment. Purified PBMCs were then resuspended in RPMI-1640 (Gibco, Carlsbad, CA, USA) medium supplemented with 10% FBS (HyClone, Logan, Utah, USA), 3 mM L-glutamine (Sigma-Aldrich, St. Louis, MO, USA), and 100 U/ml penicillin-streptomycin (Sigma-Aldrich, St. Louis, MO, USA), plated at 1 × 10^4^ cells per well in 96-well plates, and incubated at 37°C with 5% CO_2_. Following a 3 h incubation, the culture medium was replaced with a serum-free medium prior to stimulation with various PRR ligands and cytokines.

### PRR Ligand and Cytokine Treatment

Bovine (*n* = 4) and porcine (*n* = 6) PBMCs were treated with PRR ligands and cytokines, as shown (Supplementary Table 1). After 96 h, the cell culture medium (supernatant) was harvested, and cytotoxicity [via lactate dehydrogenase (LDH) release] and cell proliferation [via 5-bromo-2′-deoxyuridine (BrdU) incorporation] were assessed.

### PRR Ligand- and Cytokine-Mediated LDH Release Assay in Bovine and Porcine PBMCs

Cytotoxicity levels were detected in the supernatant of bovine and porcine PBMCs treated with PRR ligands and cytokines, as described above. An LDH release assay was performed using the CytoTox 96 Non-Radioactive Cytotoxicity Assay (Promega, Madison, WI, USA), following the manufacturer's protocol. The percentage of LDH release was calculated as follows: percentage of LDH release = 100 × (absorbance reading of treated well—absorbance reading of untreated control)/(absorbance reading of maximal LDH release control—absorbance reading of untreated control). The lysis buffer provided by the kit was used to achieve complete cell lysis, and the supernatant from the lysis buffer-treated cells was used to determine maximum LDH release control.

### BrdU Incorporation Assay in Bovine and Porcine PBMCs

The effects of PRR ligands and cytokines on the proliferation of bovine and porcine PBMCs were assessed using a BrdU Cell Proliferation Assay Kit (Cell Signaling Technology, Beverly, MA, USA) based on the incorporation of BrdU during DNA synthesis. Briefly, 10 μM BrdU was added to the cell culture and incubated for 4 h at 37°C. The cells were then fixed and incubated with an anti-BrdU mouse monoclonal antibody, followed by horseradish peroxidase-conjugated goat anti-mouse antibodies. The chromogenic substrate tetramethylbenzidine was used for color development. Absorbance was measured at a dual wavelength of 450/550 nm.

### Cattle and Pigs

In order to evaluate the potential of PRR ligands and recombinant cytokines as vaccine adjuvants and to investigate their ability to induce cellular and humoral immune responses and long-term immunity, field experiments using cattle and pigs were conducted. For the field experiment, FMD antibody-seronegative animals from 2 farmhouses were used (the cattle were 5 months old and the pigs were 10 weeks old). The cattle and pigs were divided into 3 and 4 groups, respectively (*n* = 5/group). The animals were kept in closed containments during the study. The studies were performed according to institutional guidelines, with approval from the Ethics Committee of the Animal and Plant Quarantine Agency.

### Immunization and Sampling

O/TWN/97-R Ag was used as the FMD Ag, and the vaccine composition for the positive control group was as follows: 1 ml vaccine prepared as a single dose, which included 15 μg of O/TNW/97-R Ag, ISA 206 (50%, w/w), 10% Al(OH)_3_, and 150 μg Quil-A.

Vaccination was performed twice at a 28 days interval, and 1 ml of vaccine (1 dose) was administered via the deep intramuscular route on the necks of the animals. Blood samples were collected at 0, 14, 28, 56, 84, 112, 140, and 168 dpv from cattle and at 0, 14, 28, 42, 56, 70, and 84 dpv from pigs. The animals were monitored daily for body temperature, symptoms at vaccination site, and appetite. Serum samples were stored at −80°C until tests were performed.

### Serological Assays

#### ELISA for the Detection of Structural Protein (SP) Antibodies

To detect SP antibodies in the sera, PrioCHECK FMDV type O (Prionics AG, Switzerland) was used. Absorbance in the ELISA plate was converted to a percent inhibition (PI) value. When the PI value was 50% or above, the animals were considered antibody positive.

#### Virus Neutralization Test

A virus neutralization test was performed according to the World Organization for Animal Health (OIE) manual (18). The sera were heat inactivated at 56°C for 30 min in a water bath. Cell density was adjusted to form a 70% monolayer, and 2-fold serial dilutions of sera samples (1:4–1:512) were prepared. The diluted sera samples were then incubated with a 100-tissue culture infectious dose (TCID)_50_/0.5 ml homolog virus for 1 h at 37°C. After 1 h, a LF-BK (bovine kidney) cell suspension was added to all wells. After 2–3 days, CPE was checked to determine the titers, which were calculated as Log_10_ of the reciprocal antibody dilution required to neutralize 100 TCID_50_ of the virus (19, 20).

### Statistics

All quantitative data are expressed as mean ± SEM, unless otherwise stated. Between groups, statistical significances were assessed using two-way ANOVA followed by the Bonferroni *post-hoc* test and one-way ANOVA followed by Tukey's *post-hoc* test. ^*^p < 0.05; ^**^p < 0.01; ^***^p < 0.001. Survival curves were built using the Kaplan-Meier method and differences were analyzed using the log-rank sum test. GraphPad Prism 5 (GraphPad, San Diego, CA, USA) software was used for all statistical analyses.

## Results

### Inclusion of PRR Ligands and Cytokines as FMD Vaccine Adjuvants Induce Potent Memory Responses and Elicit a Protective Effect Against FMDV Infection in Mice

Mouse experiments were performed to evaluate the potential of PRR ligands and recombinant cytokines as FMD vaccine adjuvants and the induction of adjuvant-mediated memory immune response (Figure 1A). As indicated by SP O ELISA, the group administered with PRR ligand and FMDV O Ag showed high antibody titers at 28 dpv (Figure 1B), and antibody titers were also significantly elevated in the experimental groups treated with rmIFNα (*p* < 0.001), rmIFNγ+rmIL-2+rmTNFα (*p* < 0.05), rmIL-15+rmIL-18 (*p* < 0.05), TDB+c-di-GMP (*p* < 0.05), and R848+c-di-GMP (*p* < 0.01) compared to the positive control group. Furthermore, at 58 dpv, significantly higher antibody titers were observed in all experimental groups vaccinated with cytokines and PRR ligands as adjuvants (*p* < 0.01 and *p* < 0.001, respectively). To determine the effect of the vaccination itself on body weight, mouse body weight was monitored once a week for 8 weeks (56 d) following vaccination, but no significant differences were found between the groups (Figure 1C).

Later, 56 days following vaccination, the mice were challenged with O VET 2013 and monitored for survival rates (Figure 1D) and changes in body weight (Figure 1E). Mice receiving PRR ligands and cytokines as vaccine adjuvants had a 100% survival rate without weight loss. By contrast, 100% of the mice in the negative control group died by 4 dpc, and the mice in the positive control group had a survival rate of 40%.

### PRR Ligands and Cytokines Promote the Expansion of Memory Immune Cells

**Figure 2:**
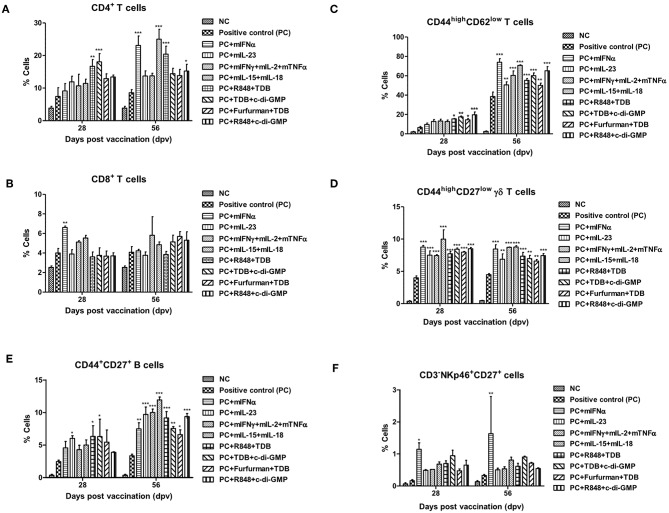
PRR ligands and cytokines promote the expansion of memory immune cells C57BL/6 mice were administered either a combination of PRR ligands or cytokines with the vaccine based on the vaccine composition of the positive control group. The PRR ligands and cytokines used in the experiment and the vaccination method are summarized in Figure 1. Peritoneal exudate cells (PEC) sampling was performed at 28 and 56 dpv for the flow cytometric assay. PEC was immunostained with fluorochrome-conjugated Abs to CD3, CD4, CD8a, CD44, CD62L, CD27, γδ TCR, CD335 (NKp46), CD11c, Anti-MHC Class II, CD11b, and anti-F4/80. Data were acquired by flow cytometry and analyzed by FlowJo software vX 0.7. **(A–E)** represent the expansion of immune cells; **(A)** CD4^+^ T cells; **(B)** CD8^+^ T cells; **(C)** CD44^high^ CD62^low^ T cells; **(D)** CD44^high^ CD27^low^ γδ T cells; **(E)** CD44^+^CD27^+^ B cells; **(F)** CD335 (NKp46)^+^CD27^+^ cells. The data are the mean ± SEM of triplicate measurements; statistical analyses were performed using two-way ANOVA with Bonferroni correction; *p < 0.05, **p < 0.01, ***p < 0.001.

To investigate cellular and humoral immune responses mediated by PRR ligands and cytokines, the expansion of immune cells was analyzed using flow cytometry. CD4^+^ T cells were expanded in the group supplemented with R848+TDB and TDB+c-di-GMP on 28 dpv and mIFN, mIL-15+mIL-18, R848+TDB, and TDB+c-di-GMP on 56 dpv (Figure 2A, Supplementary Figure 3A). CD8^+^ T cells in the mIFNα-treated group had a lower absolute cell number than CD4^+^ T cells but showed significantly higher cell expansion on 28 dpv (Figure 2B, Supplementary Figure 3B). The expansion of CD44^high^ CD62^low^ T cells, a memory T cell marker, increased rapidly at 56 dpv compared to 28 dpv. Although the cell number of these effector memory T cells increased in all the adjuvant treated groups at 56 dpv (Figure 2C, Supplementary Figure 3C), PRR ligands induced the expansion of these cells more significantly than cytokines at 28 dpv.

CD44^high^ CD27^low^ γδ T cells, known as memory γδ T cells, were expanded 8–12% by the addition of PRR ligands and cytokines, and no difference was observed between 28 and 56 dpv (Figure 2D, Supplementary Figure 3D). The expansion of CD44^+^CD27^+^ B cells, known as memory B cells, was significantly increased at 28 dpv in the mIL-23, R848+TDB, and TDB+c-di-GMP adjuvant groups. Overall, the absolute cell number was higher at 56 dpv compared to 28 dpv, and memory B cells were significantly expanded in all PRR ligand- and cytokine-supplemented groups. In particular, the mIL-23, mIFNγ+mIL-2+mTNFα, mIL-15+mIL-18, R848+TDB, and R848+c-di-GMP supplemented groups showed a significant increase in cell expansion (*p* < 0.001; Figure 2E, Supplementary Figure 3E). In the mIFNα-treated group, CD335 (NKp46)^+^CD27^+^ cells, known as memory-like NK cells, expanded as well as increased in number (Figure 2F, Supplementary Figure 3F). The populations of DCs (CD11c^+^MHC II^+^) and MΦs (CD11b^+^F4/80^+^) in this study were not significant (data not shown).

To validate FMDV O Ag-specific T cell response and amplification of memory T cell response due to Ag re-stimulation, IFNγ^pos^CD4^+^ T cells and IFNγ^pos^CD8^+^ T cells percentages were compared via flow cytometric analysis of purified T cells from pre- or post- Ag injected mouse PEC with or without Ag treatment. The percentage of IFNγ^pos^CD4^+^ T cells was significantly increased by Ag treatment, and these Ag-specific T cell responses were remarkably amplified by Ag re-stimulation in the post- Ag injected group (*p* < 0.001; Supplementary Figure 1A). A similar trend was detected in the percentage of IFNγ^pos^CD8^+^ T cells (*p* < 0.001, Supplementary Figure 1B). ELISA results demonstrated that IFNγ expression in T cell culture supernatants was also significantly increased by Ag treatment (*p* < 0.001), and the production of IFNγ was significantly enhanced by Ag re-stimulation (*p* < 0.001, Supplementary Figure 1C).

### Administration of Individual PRR Ligands, Alone or in Combination, Does Not Elicit LDH Release-Related Cytotoxicity in Bovine- and Porcine-Derived PBMCs

LDH release was examined to observe cytotoxicity due to the PRR ligands, gels, and saponins in bovine-derived PBMCs. Low cytotoxicity was observed in all treated cells (Supplementary Figure 2A). LDH release following treatment of bovine-derived PBMCs with individual PRR ligands and a vaccine-adjuvant mixture of oil+gel+saponin, is shown (Supplementary Figure 2B). At this time, no cytotoxicity due to the adjuvant mixture was observed, compared with control cells. Moreover, cells treated with either the PRR ligand alone (Supplementary Figure 2C) or PRR ligands in combination with oil+gel+saponin mixture also exhibited low LDH release levels (Supplementary Figure 2D).

When porcine-derived PBMCs treated with individual PRR ligands, or with individual PRR ligands combined with the oil+gel+saponin mixture, were assessed for LDH release, no toxicity was observed at the adjuvant concentrations used in this study, which was similar to the results obtained for bovine-derived PBMCs (Supplementary Figures 2E,F). A similar pattern was observed when porcine-derived PBMCs treated with either the PRR ligand alone or PRR ligands in combination with oil+gel+saponin mixture also exhibited low LDH release levels (Supplementary Figures 2G,H).

### Treatment With Individual PRR Ligands, Alone or in Combination, Promotes Cell Proliferation and Initiates an Immune Response in Bovine- and Porcine-Derived PBMCs

**Figure 3:**
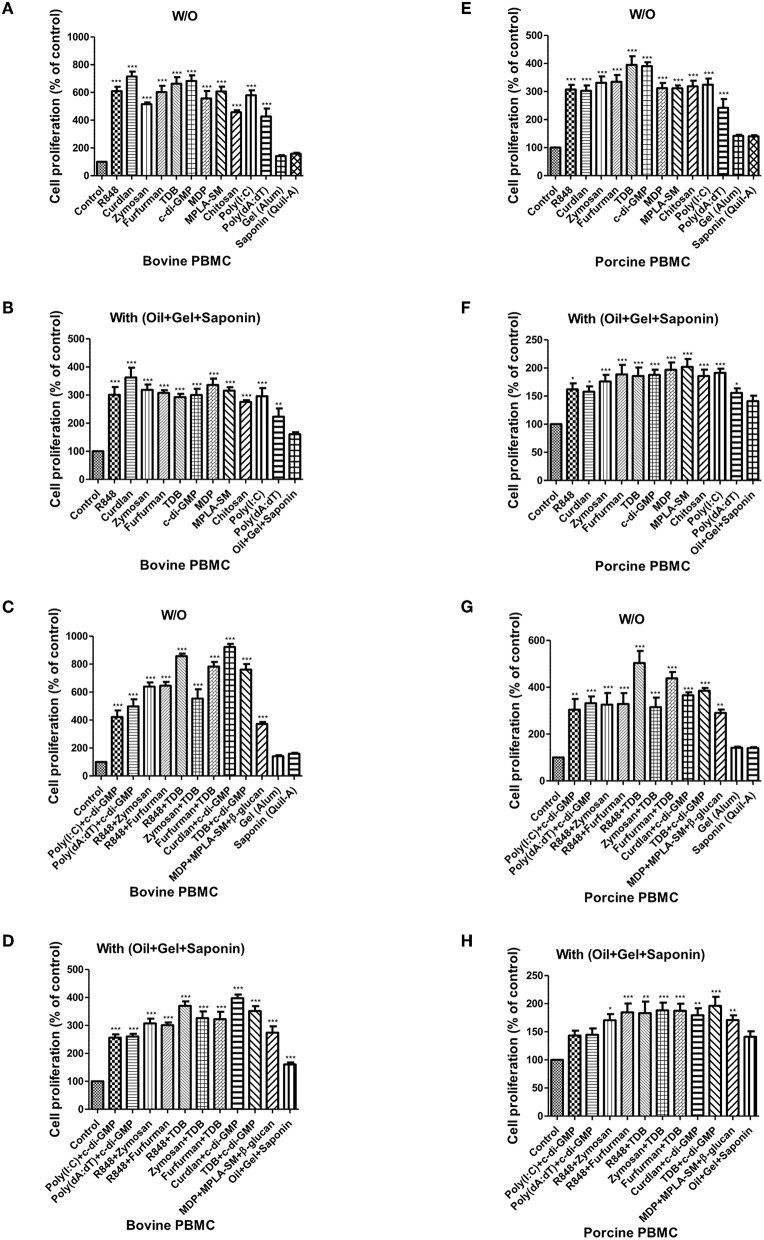
PRR ligand-induced bovine and porcine PBMC proliferation, as assessed by a BrdU cell proliferation kit. Bovine and porcine PBMCs were co-incubated with either PRR ligands alone or a combination of PRR ligands or a mixture of oil, gel, and saponin. The PRR ligands used in the experiment were as follows: R848 (TLR-7/8 agonist), Curdlan (Dectin-1 agonist), Zymosan (Dectin-2/TLR-2 agonist), Furfurman (Dectin-2 agonist), TDB (Mincle agonist), c-di-GMP (STING agonist), MDP (NOD-2 agonist), MPLA-SM (TLR-4 agonist), chitosan (NLRP3 inflammasome inducer and MR agonist), poly(I:C) (TLR-3/MDA-5 agonist), poly(dA:dT), RIG-1/CDS agonist, and AIM2 inflammasome inducer. Gel alone, saponin alone, and a mixture of oil, gel, and saponin were also tested for comparison. At specific time points (96 h) after coincubation, cell proliferation was tested using a BrdU ELISA kit. **(A–D)** represent *in vitro* cell proliferation induced by the PRRs in bovine PBMCs; **(A)** PRR ligands alone; **(B)** PRR ligands with a mixture of oil, gel, and saponin; **(C)** combination of PRR ligands; **(D)** combination of PRR ligands with a mixture of oil, gel, and saponin. **(E–H)** represent *in vitro* cell proliferation induced by the PRRs in porcine PBMCs; **(E)** PRR ligands alone; **(F)** PRR ligands with a mixture of oil, gel, and saponin; **(G)** combination of PRR ligands; **(H)** combination of PRR ligands with a mixture of oil, gel, and saponin. The data are the mean ± SEM of triplicate measurements (*n* = 6); statistical analyses were performed using one-way ANOVA with Tukey's post-test; *p < 0.05, **p < 0.01, *and* ***p < 0.001.

When bovine-derived PBMCs were treated with individual PRR ligands alone (Figure 3A) or in combination with oil+gel+saponin mix (Figure 3B), cell proliferation was observed at 96 h via BrdU incorporation, indicating that cell proliferation was increased in all PRR ligand-treated groups compared to the control group. Among these, Curdlan, TDB, c-di-GMP, R848, and Furfurman, in particular, showed the greatest effect. In addition, treatment of PBMCs with PRR ligand combinations or individual PRR ligands combined with the oil+gel+saponin mixture increased cell proliferation in all experimental groups, compared with that of the control group. However, the increase in cell proliferation was relatively lower when oil+gel+saponin was not added (W/O). The results of cell proliferation following treatment with PRR ligand combination with or without oil+gel+saponin are shown (Figures 3C,D). While cell proliferation was increased in all treatment groups compared to the control group, the experimental groups treated with R848+TDB, Furfurman+TDB, Curdlan+c-di-GMP, and TDB+c-di-GMP, in particular, showed the highest values.

The results of cell proliferation in porcine-derived PBMCs at 96 h following treatment with individual PRR ligands and cytokines are shown (Figure 3E). Increased cell proliferation was observed in all groups treated with the PRR ligands and cytokines, among which TDB and c-di-GMP, in particular, showed the greatest effect. Treatment with individual PRR ligands and cytokines combined with oil+gel+saponin increased cell proliferation in all experimental groups, compared with the control group, as was observed in bovine-derived PBMCs. However, the extent of the increase in cell proliferation was relatively lower when oil+gel+saponin was not added (Figure 3F). Cell proliferation following treatment with PRR ligand combinations, with or without oil+gel+saponin, is shown (Figures 3F,G). Compared with the control group, increased cell proliferation was observed in all treatment groups, and, in particular, the experimental groups treated with R848+TDB, Furfurman+TDB, and TDB+c-di-GMP showed the highest values. On the other hand, cross-species comparison of cell proliferation revealed that cattle showed higher cell proliferation than pigs.

### Mincle, STING, Dectin-1/2, and TLR-7/8 Signaling Amplify Robust, Long-Lasting Memory Responses by Inducing Cellular Immune Responses in the Early Stages After Vaccination in Cattle and Pigs

**Figure 4:**
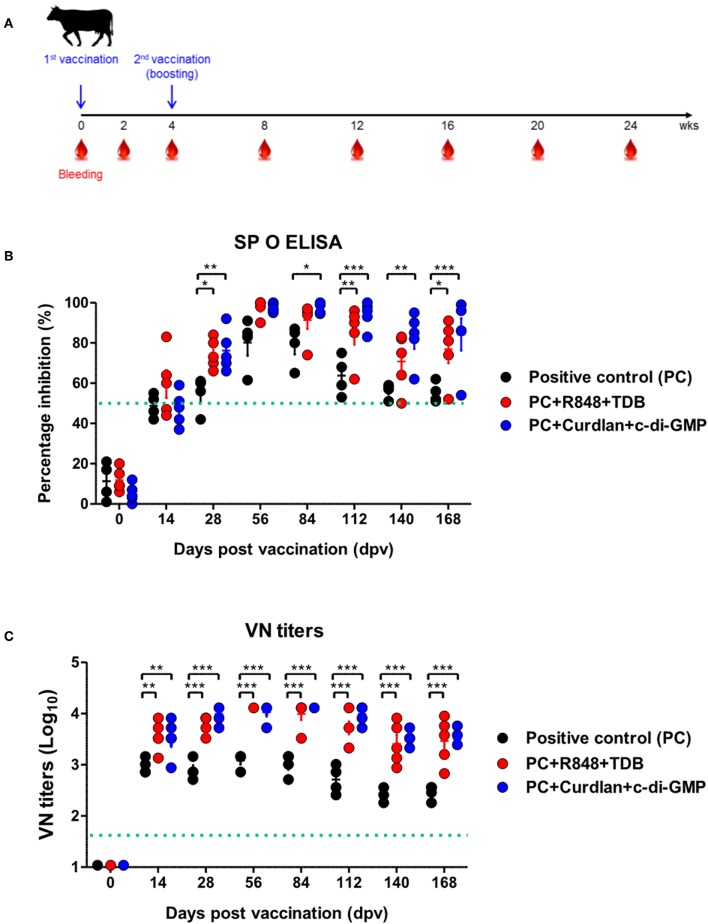
PRR ligand-mediated long-lasting memory response in cattle. Cattle were administered either a combination of R848 (TLR-7/8 agonist) and TDB (Mincle agonist) or Curdlan (Dectin-1 agonist) and c-di-GMP (STING agonist) with the vaccine, based on the vaccine composition of the positive control group. A positive control group of cattle received 15 μg (1 dose for cattle use) of O/TWN/97-R Ag, ISA 206 (50%, w/w), 10% Al(OH)_3_, and 150 μg Quil-A without PRR ligands. The vaccination was performed twice at a 28 days interval, and 1 ml vaccine (1 dose) was injected via the deep intramuscular route on the necks of the animals. Blood samples were collected at 0, 14, 28, 56, 84, 112, 140, and 168 dpv from the cattle for the serological assays. **(A–C)** represent **(A)** the strategy for this study; **(B)** antibody titers by SP O ELISA; **(C)** virus-neutralizing antibody titers. The data are the mean ± SEM of triplicate measurements; statistical analyses were performed using two-way ANOVA with Bonferroni correction; *p < 0.05, **p < 0.01, ***p < 0.001.

To evaluate the effect of the adjuvants and the memory response mediated by the PRR ligands in farm-raised cattle, R848+TDB and Curdlan+c-di-GMP (both of which showed a significant effect in the PRR ligand-screening experiment using bovine PBMCs) were applied to an animal experiment using the strategy shown in Figure 4A. At 28 dpv after the first vaccination, Ab titers were determined by SP O ELISA, and significantly higher antibody titers were observed in the experimental groups treated with R848+TDB (*p* < 0.05) and Curdlan+c-di-GMP (*p* < 0.01) than in the positive control group; the antibody titers were maintained at high levels, up to 168 dpv after boosting (*p* < 0.05 and *p* < 0.001, respectively) (Figure 4B). In addition, when the VN titer was examined, the titer was significantly higher in the PRR ligand-treated groups than in the control group from 14 dpv (*p* < 0.01), and the titer was maintained at very high levels even until 140 dpv (*p* < 0.001) (Figure 4C).

**Figure 5:**
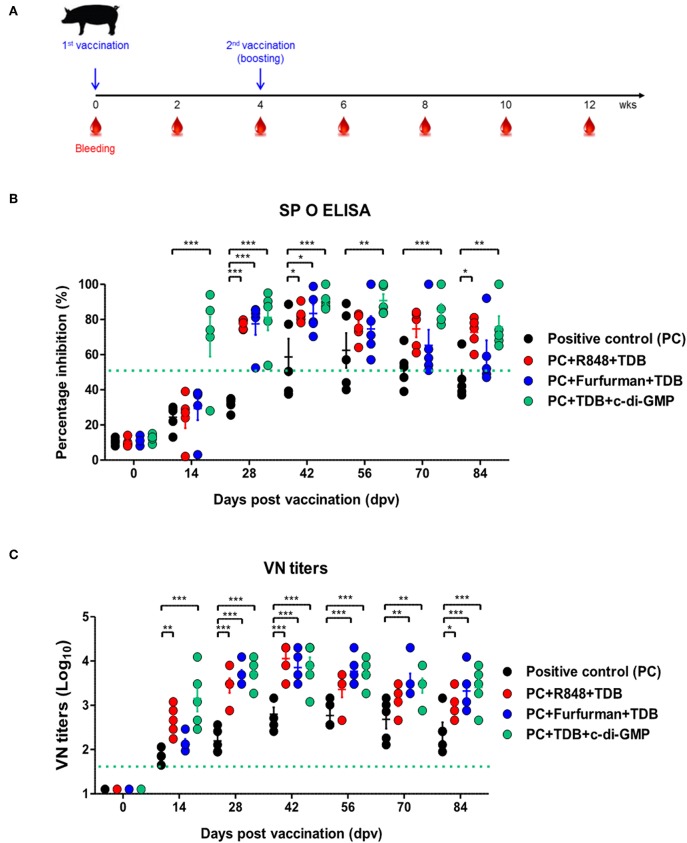
PRR ligand-mediated long-lasting memory response in pigs. Pigs were administered a combination of R848 (TLR-7/8 agonist) and TDB (Mincle agonist) or Furfurman (Dectin-2 agonist) and TDB (Mincle agonist) or TDB (Mincle agonist) and c-di-GMP (STING agonist) with the vaccine based on the vaccine composition of the positive control group. The positive control group of pigs received 15 μg (1 dose for pig use) of O/TWN/97-R Ag, ISA 206 (50%, w/w), 10% Al(OH)_3_, and 150 μg Quil-A without PRR ligands. The vaccination was performed twice at a 28 days interval, and 1 ml vaccine (1 dose) was injected via the deep intramuscular route on the necks of the animals. Blood samples were collected at 0, 14, 28, 42, 56, 70, and 84 dpv from the pigs for the serological assays. **(A–C)** represent **(A)** the strategy for this study; **(B)** antibody titers by SP O ELISA; **(C)** virus-neutralizing antibody titers. The data are the mean ± SEM of triplicate measurements; statistical analyses were performed using two-way ANOVA with Bonferroni correction; *p < 0.05, **p < 0.01, ***p < 0.001.

To investigate the effect of the adjuvants as well as the memory response mediated by the PRR ligands in farm-raised pigs, R848+TDB, Furfurman+TDB, and TDB+c-di-GMP (both of which showed a significant effect in the PRR ligand-screening experiment using porcine PBMCs) were applied to the animal experiment (Figure 5A). To determine the antibody titers induced by vaccination, SP O ELISA was performed using porcine serum. Antibody titers were significantly increased (*p* < 0.001) in the TDB+c-di-GMP-treated group at 14 dpv compared to the positive control group (*p* < 0.001), and the antibody titers were drastically increased (*p* < 0.001) in all groups treated with the PRR ligands at 28 dpv (Figure 5B). In particular, antibody titers were maintained at significantly higher levels (up to 84 dpv, *p* < 0.01) in the TDB+c-di-GMP-treated group compared to the control group. Furthermore, when the VN titer was determined, significantly higher neutralizing antibody titers were observed in the TDB+c-di-GMP (*p* < 0.001) and R848+TDB groups (*p* < 0.01) at 14 dpv compared to the positive control group. Antibody titers were also drastically increased (*p* < 0.001) in all groups treated with PRR ligands at 28 dpv (*p* < 0.001). In particular, antibody titers were maintained at high levels in the TDB+c-di-GMP-treated group (up to 84 dpv, *p* < 0.001), while the R848+TDB-treated group showed an excellent immune-boosting effect from the early (14 dpv) to middle (42 dpv) stages (*p* < 0.01 and *p* < 0.001, respectively) post vaccination, which tended to slightly decrease thereafter. In contrast, in the Furfurman+TDB group, the neutralizing antibody titers increased somewhat slowly in the early stage (up to 14 dpv) but drastically increased thereafter from 28 to 84 dpv compared to the control group (*p* < 0.01 and *p* < 0.001, respectively) (Figure 5C). However, neutralizing antibody titers increased more rapidly in cattle than in pigs, and even after boosting, neutralizing antibody titers tended to remain at higher levels in cattle than in pigs.

The above results indicate that R848 (TLR-7/8)+TDB (Mincle) and Curdlan (Dectin-1)+c-di-GMP (STING) specifically increased the cellular immune response and induced long-lasting memory responses in cattle. Similar reactions were also observed in TDB (Mincle)+c-di-GMP (STING), R848 (TLR-7/8)+TDB (Mincle), and Furfurman (Dectin-2)+TDB (Mincle).

## Discussion

FMD is classified as an acute infectious disease in cattle and pigs. It is asymptomatic in small ruminants, which can cause persistent infections, making it difficult to eradicate (17). Currently, vaccination policies are being implemented in Korea and other countries facing FMD epidemics, and, in the event of an outbreak, large-scale vaccine production is necessary to prepare for nationwide vaccination.

The innate immune response acts as the host's first line of defense against invading pathogens. Innate immune cells, particularly dendritic cells (DCs) and macrophages (MΦs), sense external microorganisms, which are recognized by PRRs via pathogen-associated molecular patterns (PAMPs) associated with the microrganisms. PRRs not only mediate the activation of innate immune cells in the presence of a danger signal, such as infection, but they also directly regulate adaptive immune responses (21). Although adjuvants have traditionally been used to boost the immunogenicity of vaccines, little is known about the host reactivity and precise mechanisms related to adjuvants contained in animal vaccines targeted toward different livestock species.

Recently, Chen et al. (9) reported that an adjuvant mixed with MDP (NOD-2 ligand), MPL (TLR-4 agonist), and β-glucan (TLR-2, TLR-4, and TLR-6 ligands) improved immune response and protection in pigs when used in combination with FMD vaccines. However, the immunological mechanism underlying the improved immunity has not yet been clearly understood. Martinez-Lopez et al. (22) reported that Mincle plays a key signaling role in microbiota sensing by stimulating the secretion of IL-23p19 and IL-6 via the Mincle-Syk axis, thereby regulating secretion of IL-17 and IL-22 from Th17 cells. In addition, Dectin-1/2 has also been reported to regulate the immune response against *Mycobacterium tuberculosis* infection by stimulating secretion of IL-23 through Syk-CARD9 signaling and the secretion of IL-17 from Th17 in DCs and MΦs (23, 24). STING is known to be involved in the antiviral activity mediated by cytosolic DNA sensing (25) and type I IFN expression through the cGAS-cGAMP-STING pathway (26, 27). TLR-7/8, the most well-known component of the TLR pathway, has also been reported to inhibit viral replication during viral infection through IFNα secretion as well as enhance mucosal immunity and systemic immune response (28, 29). However, little research had previously been conducted on the applicability of PRR ligands as an adjuvant for FMD vaccines.

A previous study by our group confirmed that the FMDV Ag-mediated activation of DCs and MΦs is induced by the stimulation of specific PRRs such as Mincle, STING, Dectin-1/2, and TLR-7/8 in mice. In addition, cytokines, such as IL-23 and IFNα (which are directly induced by FMDV Ag and expressed in DCs and MΦs), as well as the ligands that can stimulate associated PRRs were confirmed to significantly improve the protective effect of the FMD experimental vaccines against FMDV when used as a FMD vaccine adjuvant in a host.

Based on these results, this study aimed to monitor the memory response mediated by the PRR ligands and cytokines when used as an FMD vaccine adjuvant. This would likely enable development of FMD vaccine adjuvants, and vaccine compositions containing these adjuvants, which are optimized for bovine and porcine livestock species.

In mice, injections of various PRR ligands and cytokines alone or in combination as an adjuvant induced higher antibody titers in all experimental groups compared to the positive control group after a single vaccination. Although the short *in vivo* persistence of IFNα has been mentioned as a disadvantage in several papers (30, 31), the present study demonstrated that injection of IFNα as an adjuvant generated high antibody titers and that the resulting immunity persisted up to 56 dpv. The IL-23-treated group (in which the cellular immune response and host protective effect were previously demonstrated to be significantly improved), the group treated with IFNγ+IL-2+TNFα (which are both involved in T cell activation and T cell-mediated cellular/humoral immune responses) and the group treated with IL-15+IL-18 (which are involved in mucosal immunity) all continuously maintained high antibody titers. The PRR ligand-treated groups also showed similar patterns, confirming that these adjuvants can effectively induce memory responses.

When the effect of vaccination itself on weight change was examined, a slight weight loss was observed in the IFNγ+IL-2+TNFα combination group at 7 dpv, but no significant difference was found. This may be due to the slightly higher dose of the recombinant cytokines (15 μg in total) in this group compared to that of the other groups (5–10 μg in total). Notably, Ag-specific T cell response was significantly amplified by Ag re-stimulation. Inclusion of PRR ligands and cytokines as adjuvants promoted memory T cells, memory γδ T cells, memory B cells, and memory-like NK cell expansion, thereby effectively enhancing cellular immunity and humoral immunity. The expansion of these memory cells will play an important role in host defense by enabling a more rapid and strong response to the pathogen during FMDV infection.

Taken together, the induction of robust memory responses and expansion of memory cells by PRR ligands and cytokines resulted in a complete protective effect against FMDV infection in all experimental groups (Figures 1, 2).

Zhou et al. (8) reported that R848 and poly(I:C) injected in combination with Al(OH)_3_ as an FMD vaccine adjuvant enhanced the immune response in mice. Additionally, Du et al. (32) recently showed that CVC 1302 (MDP, MPL, and β-glucan) could be added to commercially available inactivated FMDV (serotype O) vaccines as an adjuvant-induced, long-term humoral immunity in mice through the stimulation of T follicular helper cells and the germinal center response. However, these studies were performed using specific PRR ligands, and a study to investigate the protective effects of these substances against actual FMDV infection in mice had not been previously conducted. Therefore, the results of the present study, which investigated the effects of a wide range of PRR ligands and cytokines on the induction of memory response and ability to defend against FMDV (serotype O) infection, can be interpreted as a highly efficient adjuvant screening system. This screening system may be of us in a pre-animal experimental step targeting specific animal species, such as cattle and pigs, and thus can also be used to provide basic data for developing FMD vaccines via a new strategy.

Pigs are known to have lower persistence and efficacy in their immune responses compared to cattle (7). To propose FMD vaccine compositions with superior efficacy optimized for each livestock species and to overcome the immunogenicity gap between the two species, screening was performed to identify adjuvants that could stimulate the immune response specifically in cattle and pigs. In order to facilitate screening, PBMCs isolated from the whole blood of cattle and pigs were treated with various PRR ligands and cytokines alone or in combination, and the LDH release-related cell cytotoxicity and cell proliferation were examined. PBMCs consist of lymphocytes (T cells, B cells, and NK cells), monocytes, and dendritic cells. PBMCs are broadly used in the fields of immunology, infectious disease, vaccine development, and transplant immunology, among others. Therefore, bovine and porcine PBMCs are useful model systems for the study of FMD vaccine and adjuvants. No cytotoxicity was observed in any of the adjuvant concentrations used in this study, indicating that these adjuvants are safe to administer to a host. These adjuvants significantly increased cell proliferation compared to the control group, and R848+TDB and Curdlan+c-di-GMP were effective in cattle, while R848+TDB, Furfurman+TDB, and TDB+c-di-GMP were effective in pigs. These PRR ligands mediated cell proliferation in bovine and porcine PBMCs, which is expected to simultaneously stimulate various immune cells to induce cellular immune responses more efficiently. In the cross-species comparison in particular, the immune response in bovine-derived PBMCs was significantly higher than that of porcine-derived PBMCs for most adjuvants, suggesting that bovine immune cells are more sensitive to external stimuli than porcine immune cells. Our group previously identified the fundamental difference in the FMDV Ag-mediated immune response between bovine and porcine immune cells. In the previous study, even though the Ag was a porcinophilic virus, FMDV (serotype O) Ag stimulated remarkably higher cell proliferation in bovine immune cells (PBMCs, lymphocytes, monocytes, and T cells) than in porcine immune cells. The discovery of this difference may explain the phenomenon of the lower immunogenicity observed in pigs, as compared to cattle, and suggest a key clue to overcoming this problem. In addition, based on the results of treating PBMCs with a mixture of ISA 206, Al(OH)_3_, and saponin, it is expected that when these adjuvants are injected as an actual vaccine component, the oil emulsion will allow the Ag and adjuvant to be released slowly *in vivo*, thus enabling continuous stimulation of immune responses (Supplementary Figure 1, Figure 3).

Based on the findings from the screening of PRR ligands and cytokines in PBMCs isolated from each species, experimental vaccines were created, and their immunogenicity was tested in farm raised cattle and pigs. The results showed that the neutralizing antibody titers significantly increased in both the bovine and porcine groups treated with species-specific PRR ligands at 14 dpv after the first vaccination compared to the positive control group. Long-lasting immune responses were also observed after the second vaccination (boosting). The high level of titers of neutralizing antibodies confirmed in both the cattle and pigs can be interpreted to mean that the combination of ligands, such as Mincle, STING, TLR-7/8, and Dectin-1, stimulates extrinsic and intrinsic pathways simultaneously to effectively initiate innate and cellular immune responses and activate various kinase pathways, effector molecules, and transcription factors to induce cytokine secretion. The cellular immune response is there by enhanced, and the humoral immune response is strongly induced as well. In addition, the second booster vaccination at 28 dpv showed to effectively induce “recall stimulation” among the immune cells stimulated by the first vaccination, and it also plays a role in maintaining long-lasting immune responses. The combination of the Mincle+STING ligands in pigs specifically resulted in an excellent increase of the antibody titers from 14 dpv, which is expected to help overcome the disadvantages of commercial vaccines for pigs (Figures 4, 5). In addition, further studies on the efficacy of cytokine adjuvants (which showed a strong memory response-inducing effect and a protective effect in mice) should be conducted on the target animals (cattle and pigs), and the economic feasibility of the vaccine with adjuvant addition should be considered in the future.

In summary, the novel FMD vaccine platform utilizing the Mincle, STING, Dectin-1/2, and TLR-7/8 ligands as adjuvants is expected to open the door to a new era in the field of FMD prevention and treatment.

## Data Availability Statement

All datasets generated for this study are included in the manuscript/Supplementary Files.

## Ethics Statement

The animal study was reviewed and approved by The Ethics Committee of the Animal and Plant Quarantine Agency.

## Author Contributions

ML designed the research, performed and analyzed overall experiments, and wrote the manuscript. HJ supported overall experiment. SS supported animal experiment. S-MK advised experiments. BK advised the study and reviewed the manuscript. HS provide whole blood from cattle and pigs for *in vitro* study. J-HP supervised and reviewed the manuscript.

### Conflict of Interest

The authors declare that the research was conducted in the absence of any commercial or financial relationships that could be construed as a potential conflict of interest.
