# Supplementary material for: Mincle and STING-Stimulating Adjuvants Elicit Robust Cellular Immunity and Drive Long-Lasting Memory Responses in a Foot-and-Mouth Disease Vaccine
Source: Front Immunol. 2019 Oct 29;10:2509. doi: 10.3389/fimmu.2019.02509 (PMC6828931; doi:10.3389/fimmu.2019.02509)
Supplement: Supplementary file 1 [file Data_Sheet_1.pdf]

Supplementary Material

Supplementary Tables

Supplementary Table 1. List of adjuvants containing PRRs ligands, cytokine and/or oil emulsion (ISA206), Gel ($Al(OH_3)$), Saponin (Quil-A) induced cell stimulation on bovine and porcine PBMCs.

Group	Adjuvant	Dose (μg/ml)	Group	Adjuvant	Dose (μg/ml)
1	Con	-	13	Poly(I:C) (TLR-3)+c-di-GMP (STING)	(5μg+2μg)/ml
2	R848 (TLR-7/8)	10μg/ml	14	Poly (dA:dT) (RIG-I & CDS)+c-di-GMP (STING)	(5μg+2μg)/ml
3	Curdlan (DECTIN-1)	100μg/ml	15	R848 (TLR-7/8)+Zymosan (DECTIN-2/TLR-2)	(5μg+5μg)/ml
4	Zymosan (DECTIN-2/TLR-2)	10μg/ml	16	R848 (TLR-7/8)+Furfuman (DECTIN-2)	(5μg+5μg)/ml
5	Furfurman (DECTIN-2)	10μg/ml	17	R848 (TLR-7/8)+TDB (MINCLE)	(5μg+5μg)/ml
6	TDB (MINCLE)	10μg/ml	18	Zymosan (DECTIN-2/TLR-2)+TDB (MINCLE)	(5μg+5μg)/ml
7	c-di-GMP (STING)	4μg/ml	19	Furfurman (DECTIN-2)+TDB (MINCLE)	(5μg+5μg)/ml
8	MDP (NOD-2)	10μg/ml	20	Curdlan (DECTIN-1)+c-di-GMP (STING)	(50μg+2μg)/ml
9	MPLA-SM (TLR-4)	1μg/ml	21	TDB (MINCLE)+c-di-GMP (STING)	(5μg+2μg)/ml
10	Chitosan (NLRP3 inflammasome, MR)	100μg/ml	22	MDP (NOD-2) +MPLA-SM (TLR-4)+Curdlan (DECTIN-1))	(10μg+1μg+100μg)/ml
11	Poly(I:C) (TLR-3)	10μg/ml	23	Gel ($Al(OH)_3$)	10%
12	Poly(dA:dT) (RIG-1 & CDS)	10μg/ml	24	Saponin (Quil-A)	2.5μg/ml
			25	Oil (ISA206) + Gel ($AlOH)_3$) + Saponin (Quil-A)	(25%+10%+2.5μg)/ml

Supplementary Figures

Supplementary Figure 1. FMDV O Ag specific-T cells response and enhanced INFγ secretion in CD4$^+$ T cells and CD8$^+$ T cells by restimulation of FMDV O Ag.

(A)

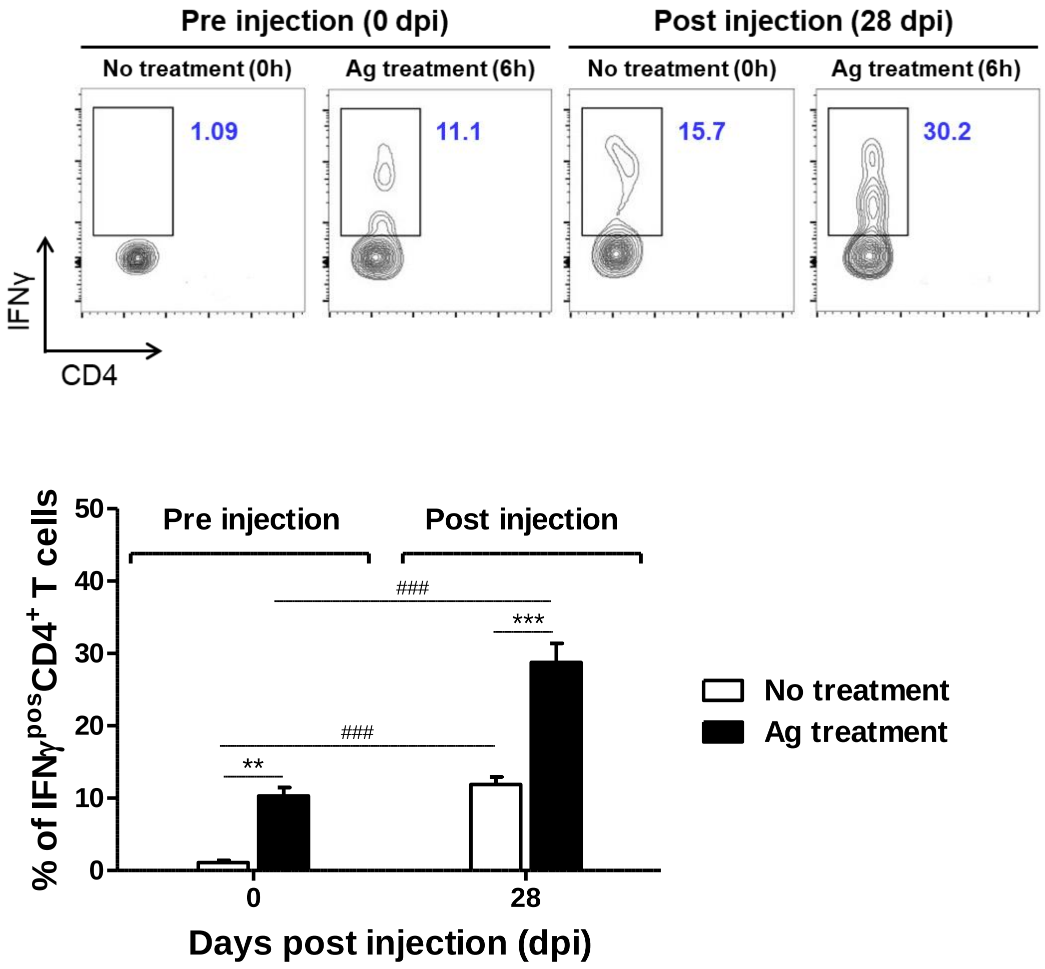

(B)

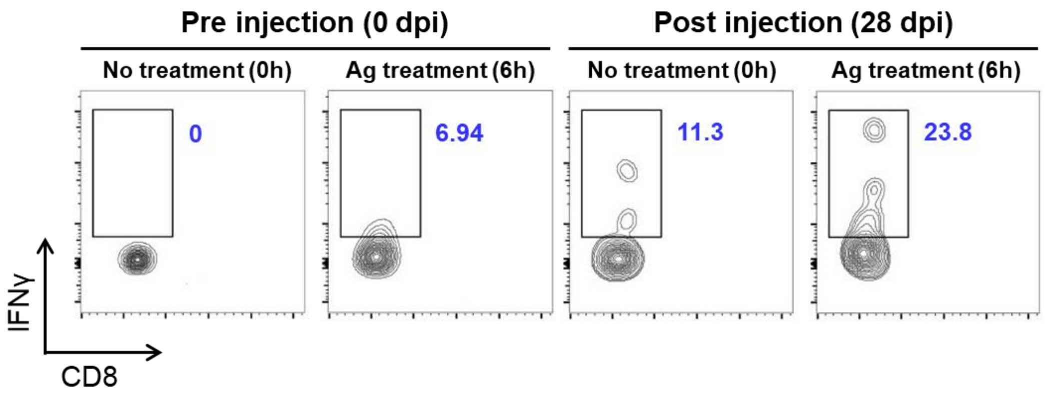

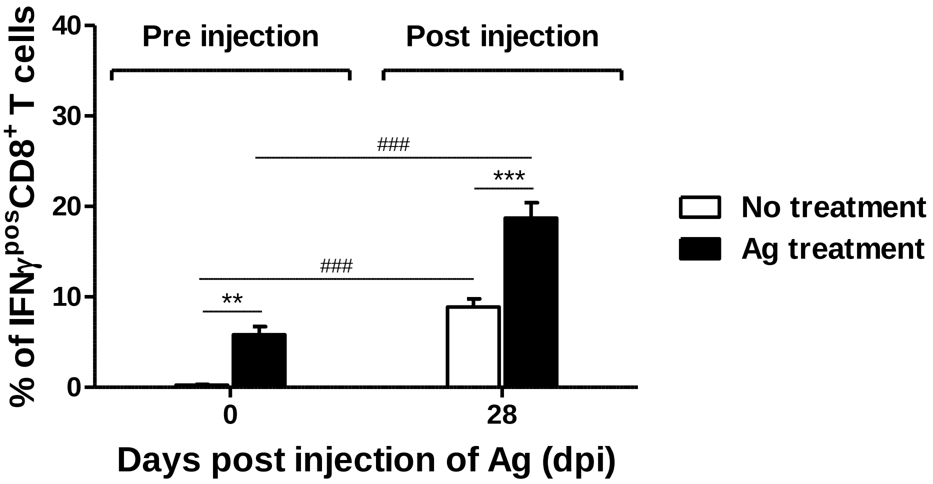

(C)

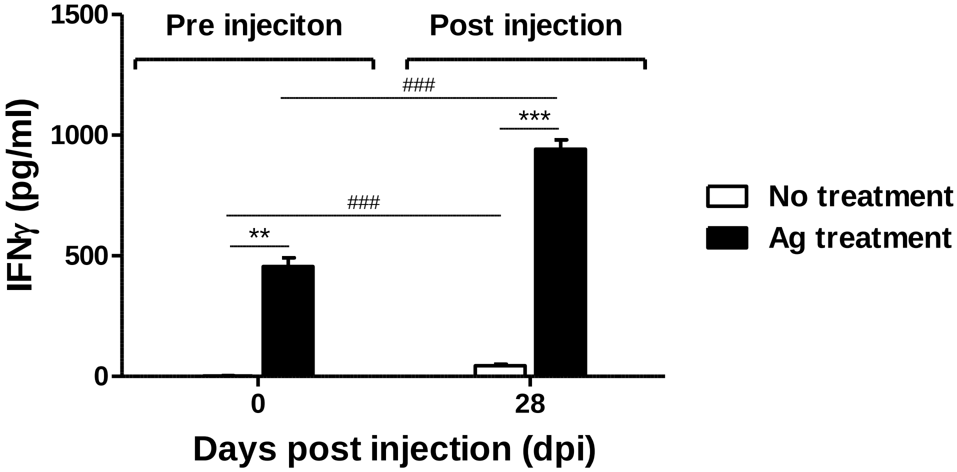

In order to identify whether FMDV O Ag-specific T cell responses and memory T cell responses were amplified by Ag re-stimulation, we isolated pre (0 dpi) and post Ag injection (28 dpi) mice PEC (PC group). T cells were purified from isolated PEC (Pan T Cell Isolation Kit II, Miltenyi Biotec) and sorted via flow cytometry (purity> 98%). T cells were cultured at 37℃ and 5% CO_2 in complete RPMI media (Gibco, Carlsbad, CA, USA) supplemented with 10% FBS (HyClone, Logan, Utah, USA), 10 mM HEPES (Gibco), 10 U/ml penicillin/streptomycin (Sigma-Aldrich), and 50 mM 2-mercaptoethanol (Sigma-Aldrich). Cells were subsequently treated *in vitro* without or with Ag (1 μg/ml) for 6 h. The percentage of $IFN\gamma^{pos}CD4^{+}$ T cells and $IFN\gamma^{pos}CD8^{+}$ T cells was compared via flow cytometry as described in 2.5. ELISA for IFNγ (R&D Systems, Minneapolis, MN, USA) was also performed on T cell culture supernatants according to the manufacturer's instructions. Panels A-E represent the FMDV O Ag specific-T cells response and enhanced INFγ secretion in $CD4^{+}$ T cells and $CD8^{+}$ T cells by restimulation of FMDV O Ag; (A) Representative FACS plot and quantitative data for percentage of $IFN\gamma^{pos}CD4^{+}$ T cells ; (B) Representative FACS plot and quantitative data for

percentage of IFNγ^{pos}CD8^{+} T cells; (C) Expression level of IFNγ in Ag treated T cell cultured supernatant.

The data are the mean±SEM of triplicate measurements (n=5/group); statistical analyses were performed using one-way ANOVA with Tukey's post test; ***p*<0.01 and $^{\#\#\#,}$ ****p*<0.001.

Supplementary Figure 2. PRR ligand-induced bovine and porcine PBMCs, as assessed by LDH release.

(A)

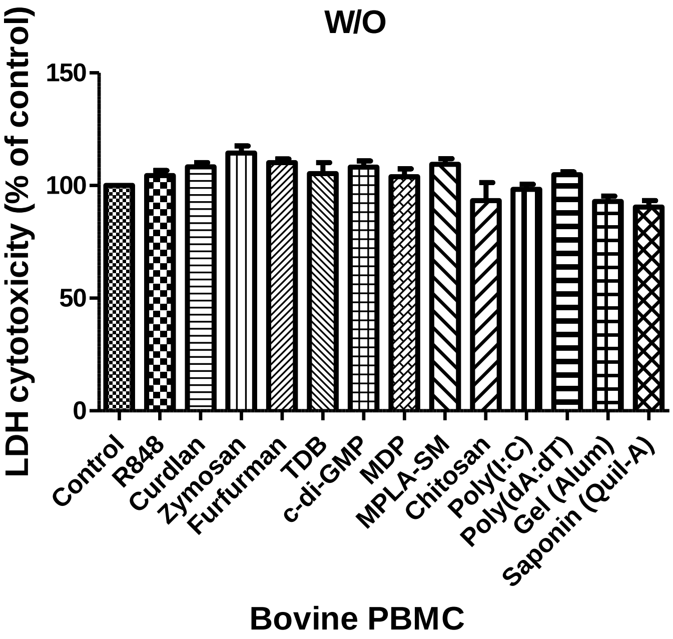

(B)

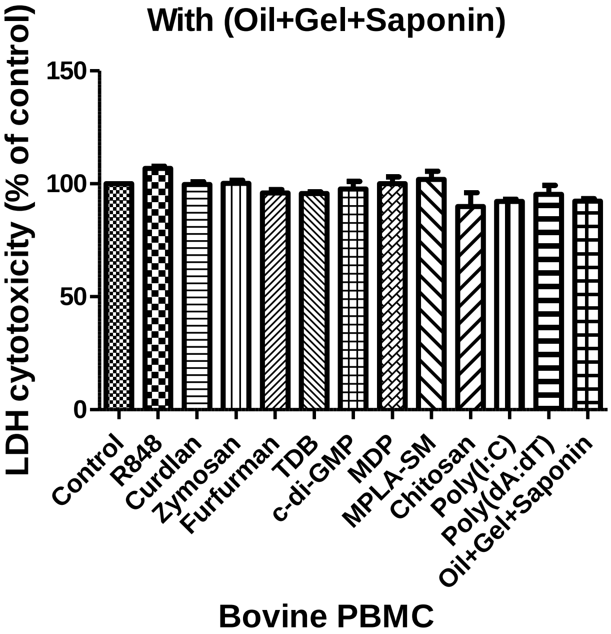

(C)

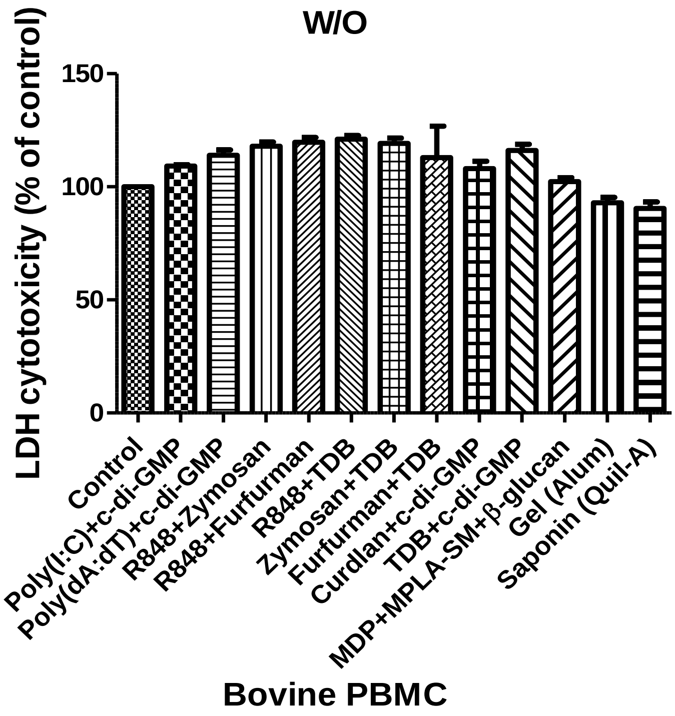
W/O
LDH cytotoxicity (% of control)
150
100
50
0
Control
Poly(I:C)+c-di-GMP
Poly(dA:dT)+c-di-GMP
R848+Zymosan
R848+Furfurman
R848+TDB
Zymosan+TDB
Furfurman+TDB
Curdlan+c-di-GMP
TDB+c-di-GMP
MDP+MPLA-SM+β-glucan
Gel (Alum)
Saponin (Quil-A)
Bovine PBMC

(D)

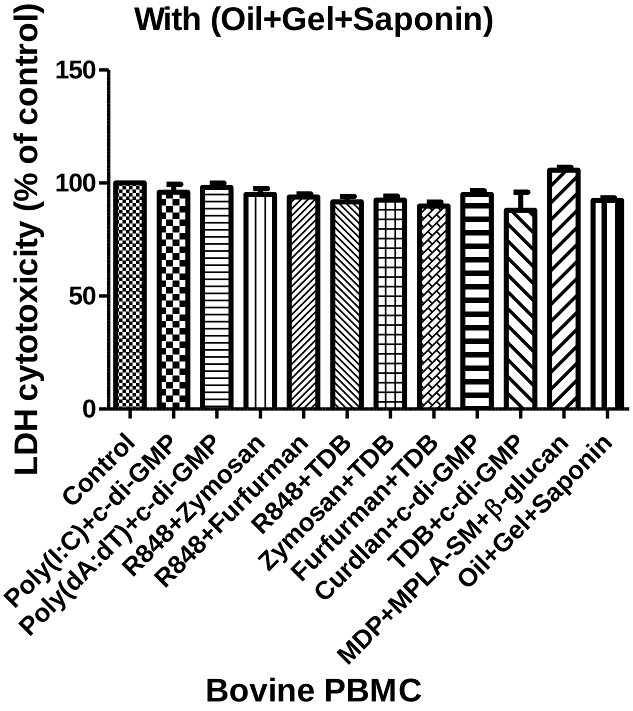
With (Oil+Gel+Saponin)
LDH cytotoxicity (% of control)
150
100
50
0
Control
Poly(I:C)+c-di-GMP
Poly(dA:dT)+c-di-GMP
R848+Zymosan
R848+Furfurman
R848+TDB
Zymosan+TDB
Furfurman+TDB
Curdlan+c-di-GMP
TDB+c-di-GMP
MDP+MPLA-SM+β-glucan
Oil+Gel+Saponin
Bovine PBMC

(E)

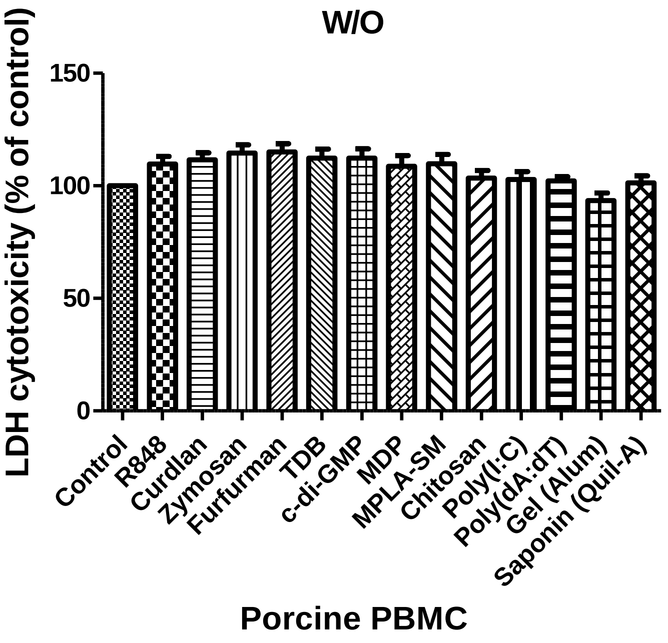
W/O
LDH cytotoxicity (% of control)
150
100
50
0
Control
R848
Curdlan
Zymosan
Furfurman
TDB
c-di-GMP
MDP
MPLA-SM
Chitosan
Poly(I:C)
Poly(dA:dT)
Gel (Alum)
Saponin (Quil-A)
Porcine PBMC

(F)

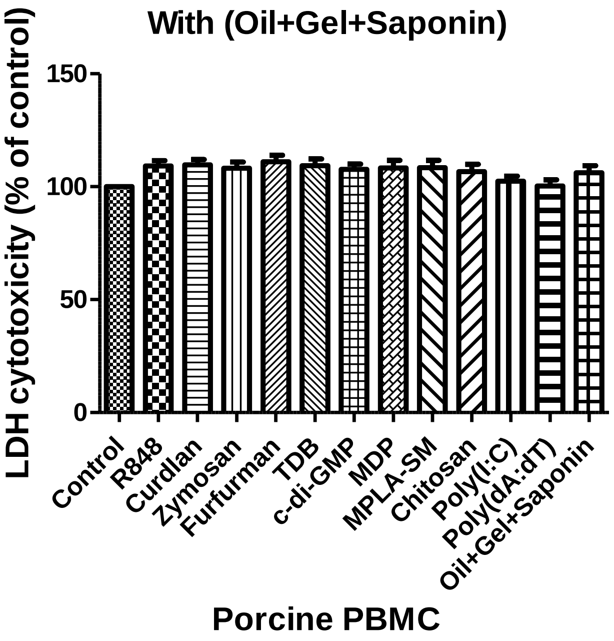
With (Oil+Gel+Saponin)
LDH cytotoxicity (% of control)
150
100
50
0
Control
R848
Curdlan
Zymosan
Furfurman
TDB
c-di-GMP
MDP
MPLA-SM
Chitosan
Poly(I:C)
Poly(dA:dT)
Oil+Gel+Saponin
Porcine PBMC

(G)

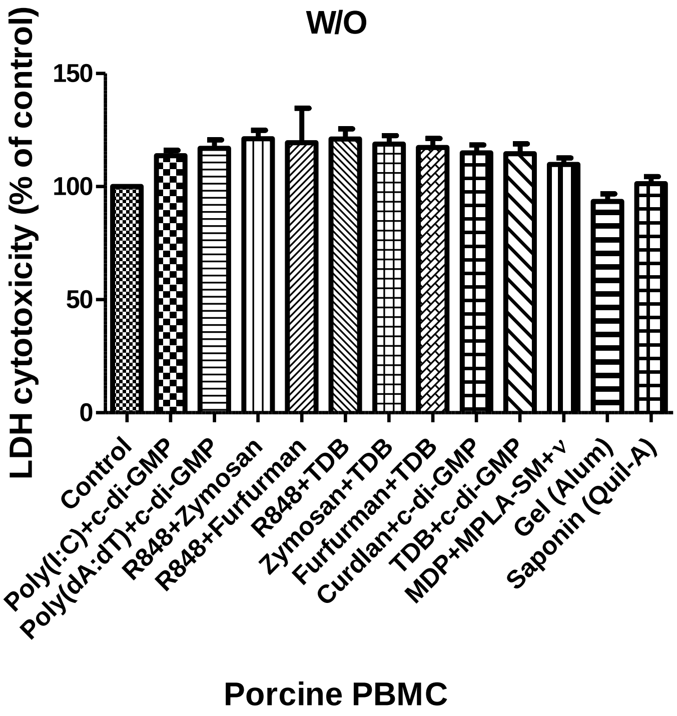
W/O
LDH cytotoxicity (% of control)
150
100
50
0
Control
Poly(I:C)+c-di-GMP
Poly(dA:dT)+c-di-GMP
R848+Zymosan
R848+Furfurman
R848+TDB
Zymosan+TDB
Furfurman+TDB
Curdlan+c-di-GMP
TDB+c-di-GMP
MDP+MPLA-SM+ν
Gel (Alum)
Saponin (Quil-A)
Porcine PBMC

(H)

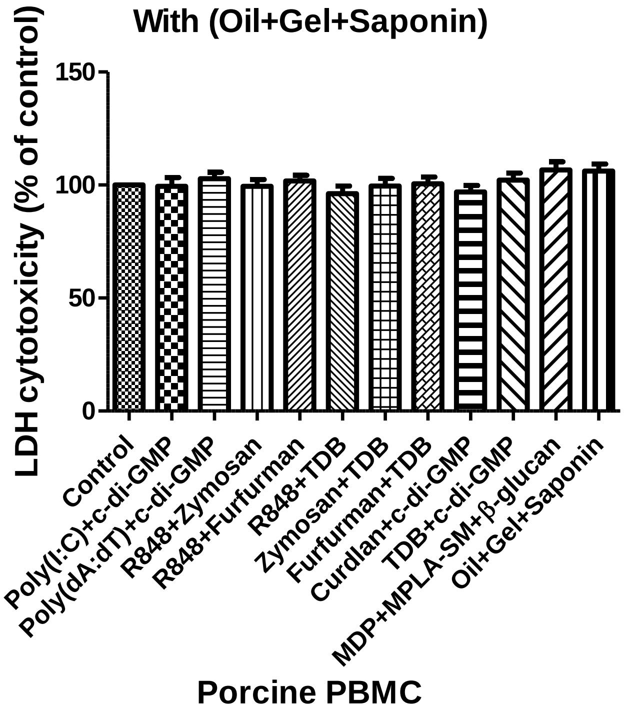
With (Oil+Gel+Saponin)
LDH cytotoxicity (% of control)
150
100
50
0
Control
Poly(I:C)+c-di-GMP
Poly(dA:dT)+c-di-GMP
R848+Zymosan
R848+Furfurman
R848+TDB
Zymosan+TDB
Furfurman+TDB
Curdlan+c-di-GMP
TDB+c-di-GMP
MDP+MPLA-SM+β-glucan
Oil+Gel+Saponin
Porcine PBMC

Bovine and porcine PBMCs were coincubated with either PRR ligands alone or with a combination of PRR ligands or a mixture of oil, gel, and saponin. The PRR ligands used in the experiment were as follows: R-848 (TLR-7/8 agonist), Curdlan (Dectin-1 agonist), Zymosan (Dectin-2/TLR-2 agonist), Furfurman (Dectin-2 agonist), TDB (Mincle agonist), c-di-GMP (STING agonist), MDP (NOD-2 agonist), monophosphoryl lipid A from *Salmonella minnesota* R595 (MPLA-SM, TLR-4 agonist), chitosan (NLRP3 inflammasome inducer and MR agonist), polyinosinic-polycytidylic acid (poly[I:C], TLR-3/MDA-5 agonist), poly(deoxyadenylic-deoxythymidylic) acid (poly[dA:dT], RIG-1/CDS agonist), and AIM2 inflammasome inducer). Gel alone, saponin alone and a mixture of oil, gel, and saponin were also tested for comparison. At specific time points (96 h) after coincubation, cytotoxicity with LDH release was tested using a CytoTox 96 Non-Radioactive Cytotoxicity Assay kit. Panels A–D represent the *in vitro* LDH release induced by the PRRs in the bovine PBMCs; (A) PRR ligand alone; (B) PRR ligand with a mixture of oil, gel, and saponin; (C) combination of PRR ligands; (D) combination of PRR ligands with a mixture of oil, gel, and saponin. Panels E–H represent *in vitro* LDH release induced by PRRs in porcine PBMCs; (E) PRR ligands alone; (F) PRR ligands with a mixture of oil, gel, and saponin; (G) combination of PRR ligands; (H) combination of PRR ligands with a mixture of oil, gel, and saponin.

The data are the mean±SEM of triplicate measurements (n=4 for bovine PBMCs, n=6 for porcine PBMCs); statistical analyses were performed using one-way ANOVA with Tukey’s post test.

Supplementary Figure 3. PRR ligands and cytokines promote the expansion of memory immune cells.

(A)

28dpv

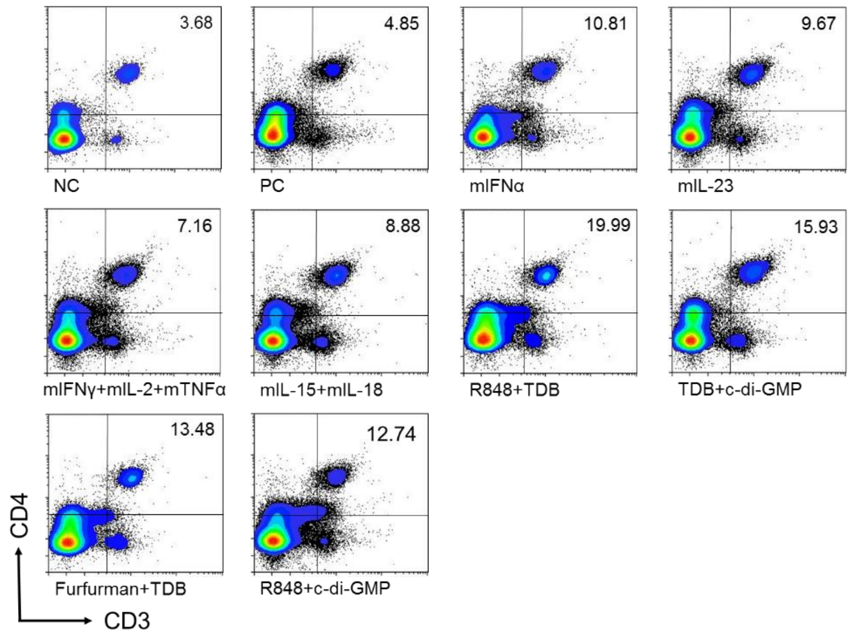

56dpv

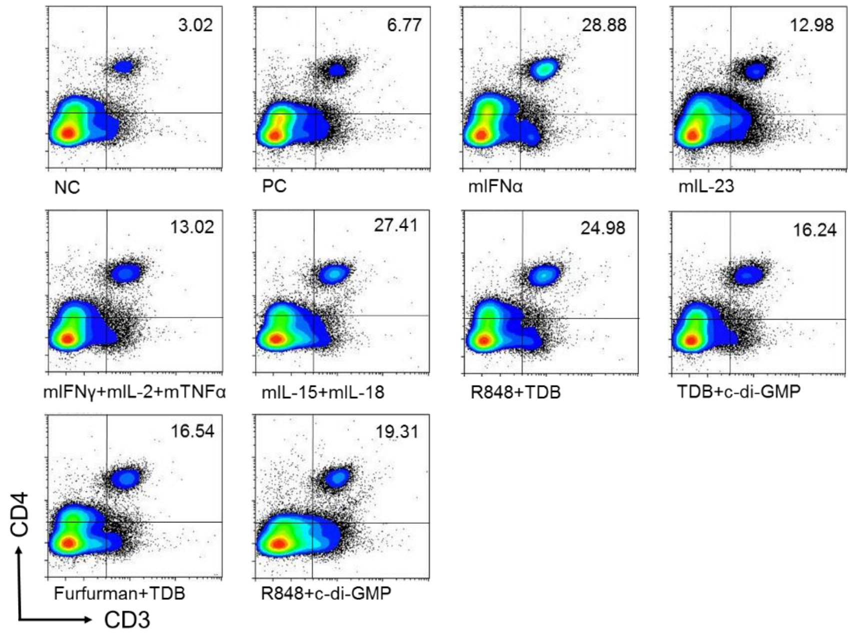

(B)

28dpv

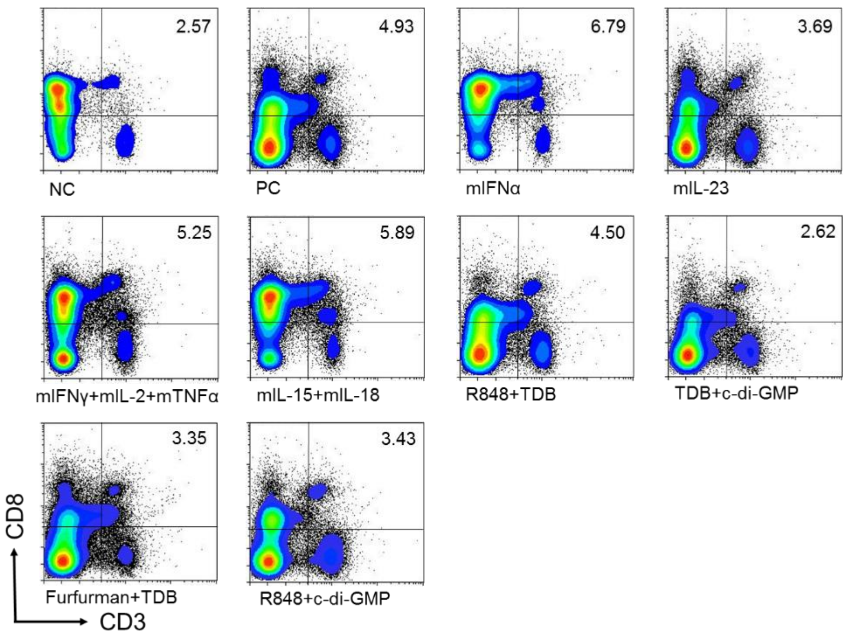

56dpv

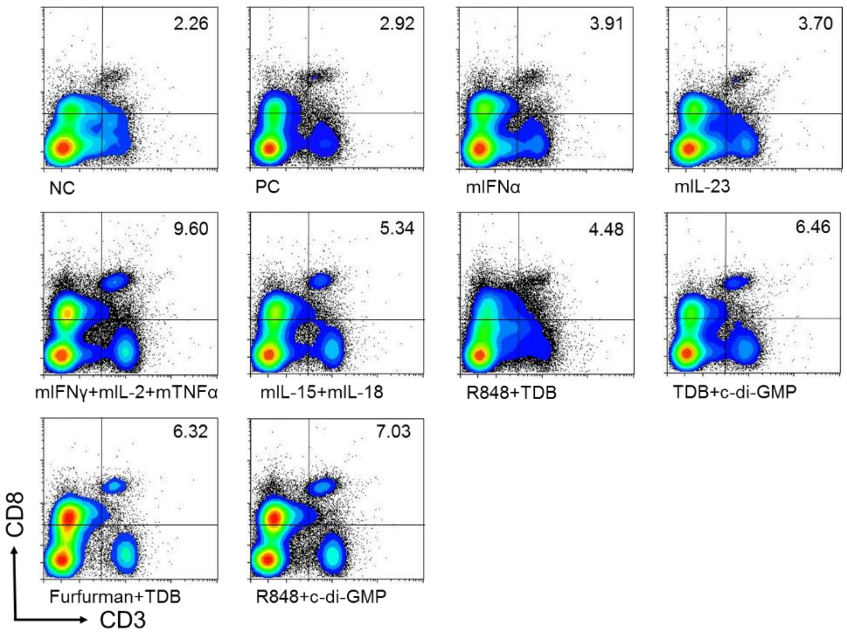

(C)

28dpv

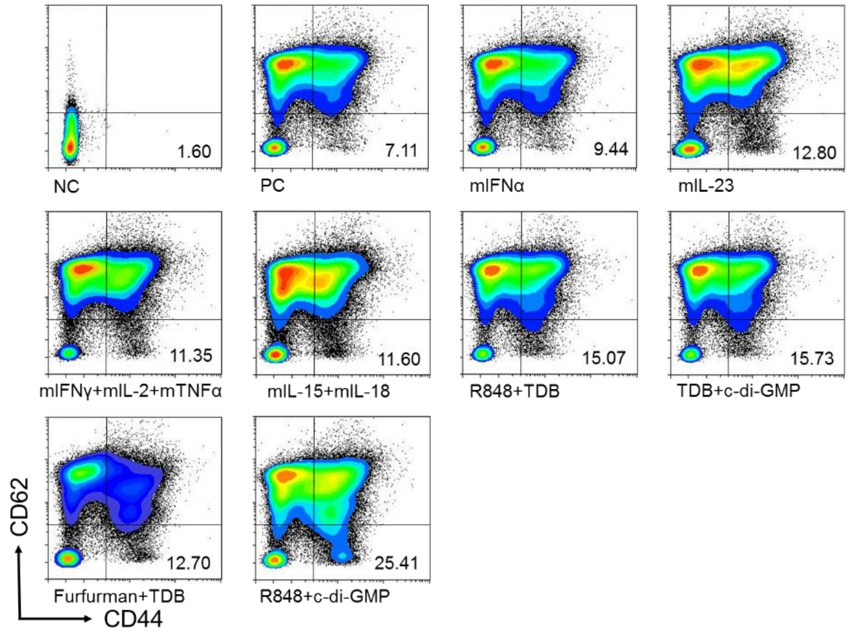

56dpv

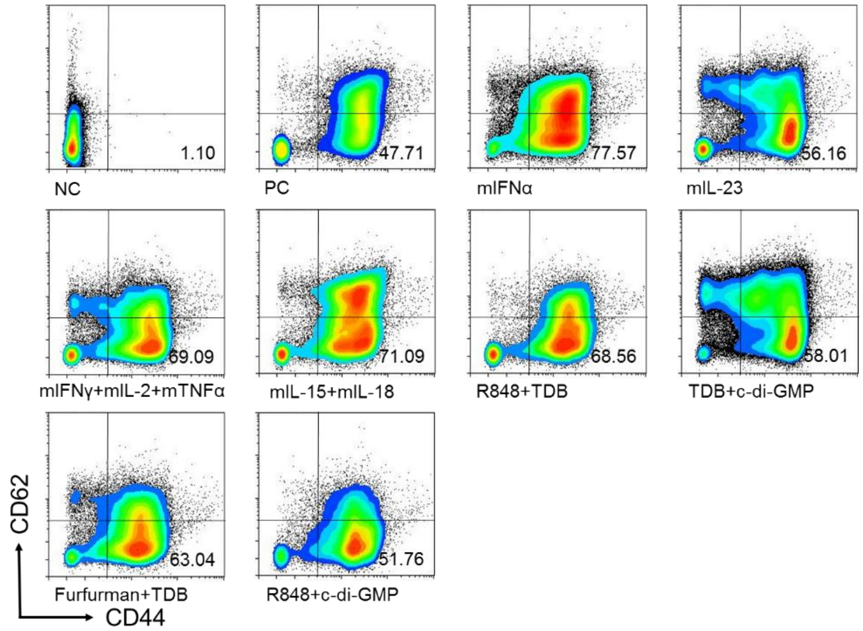

(D)

28dpv

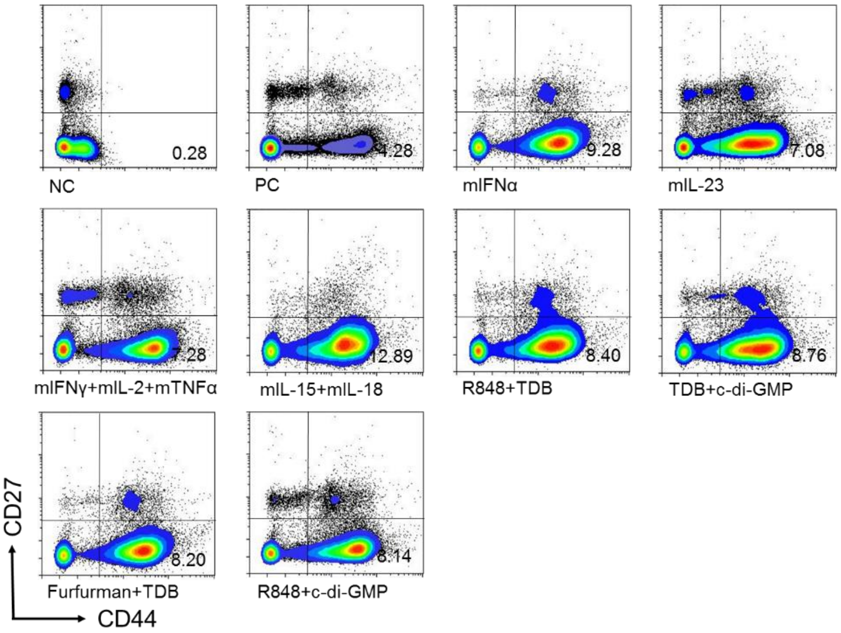

56dpv

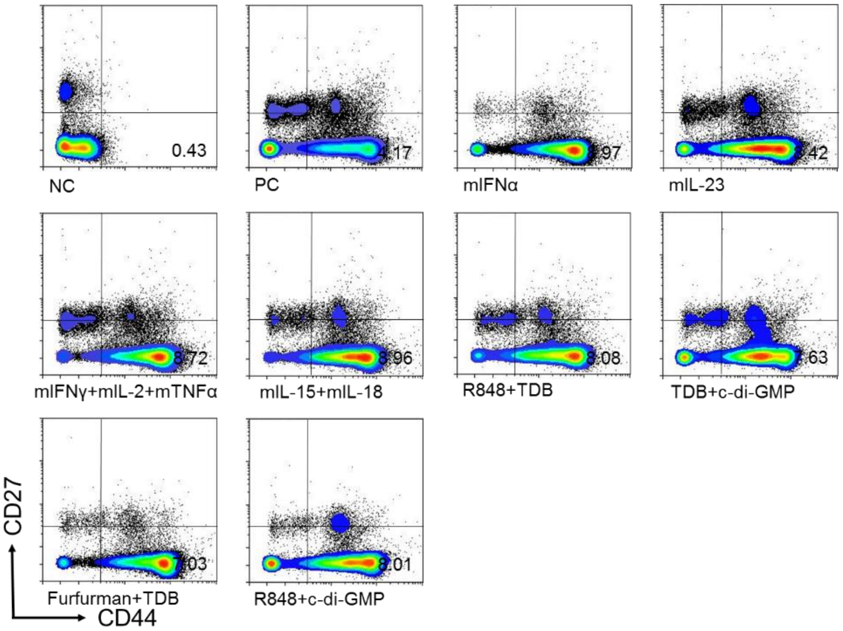

(E)

28dpv

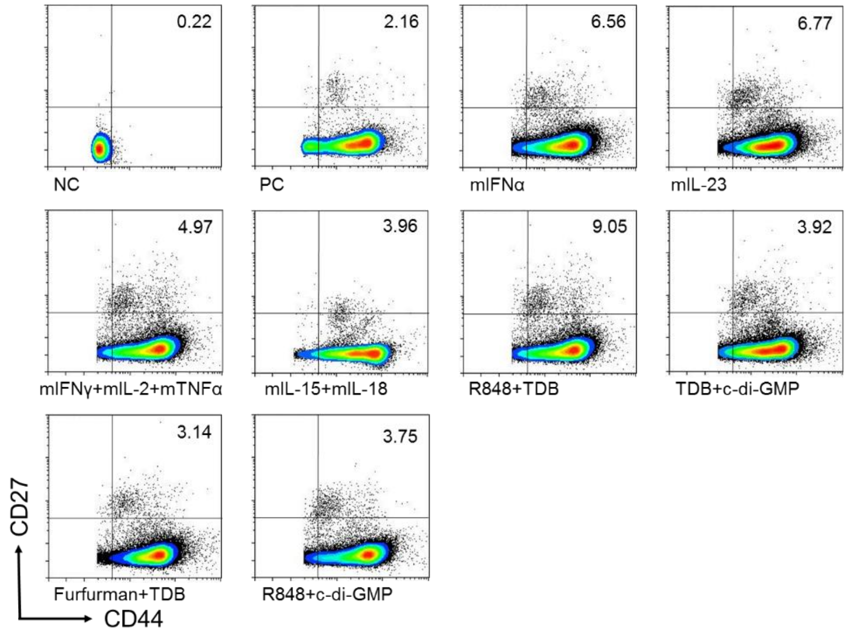

56dpv

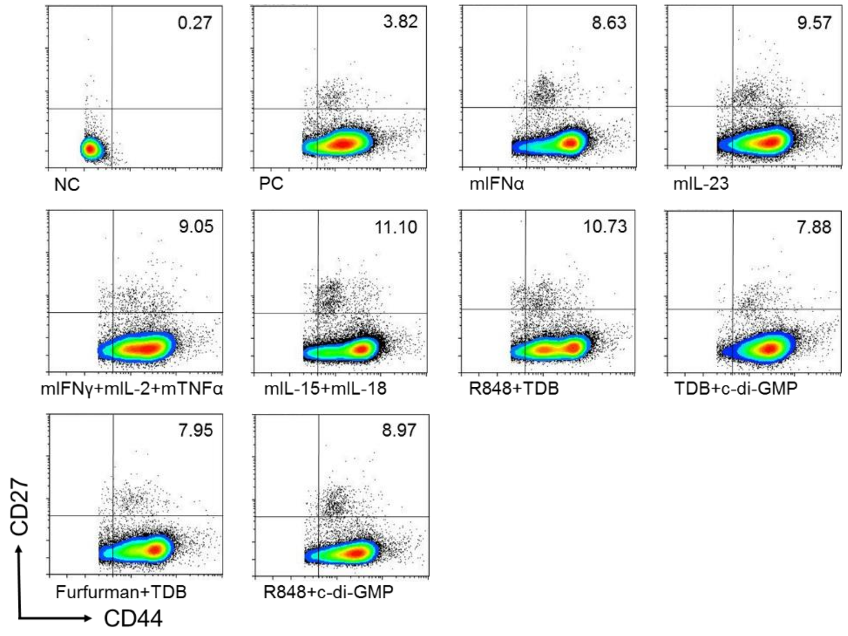

(F)

28dpv

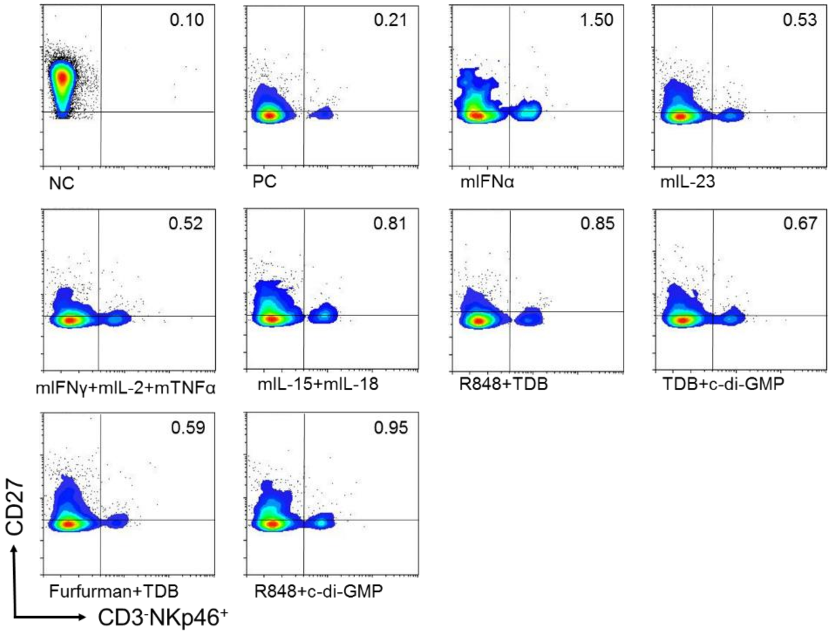

56dpv

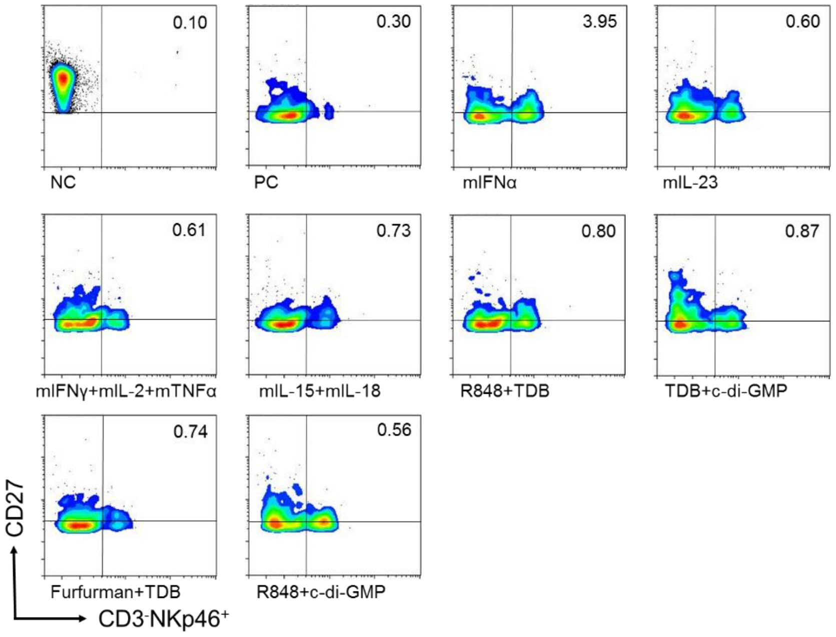

C57BL/6 mice were administered either a combination of PRR ligands or cytokines with the vaccine based on the vaccine composition of the positive control group. The PRR ligands and cytokines used in the experiment and the vaccination method are summarized in Figure 1. Peritoneal exudate cells (PEC) sampling was performed at 28 dpv and 56 dpv for the flow cytometric assay. PEC was immunostained with fluorochrome-conjugated Abs to CD3, CD4, CD8a, CD44, CD62L, CD27, γδ TCR, CD335 (NKp46), CD11c, Anti-MHC Class II, CD11b and anti-F4/80. Data were acquired by flow cytometry and analyzed by FlowJo software vX 0.7. Panels A-E represent the expansion of immune cells; (A) $CD4^{+}$ T cells; (B) $CD8^{+}$ T cells; (C) $CD44^{high}$ $CD62^{low}$ T cells; (D) $CD44^{high}$ $CD27^{low}$ γδ T cells; (E) $CD44^{+}CD27^{+}$ B cells; (F) CD335 $(NKp46)^{+}CD27^{+}$ cells.
